# The genetic connectedness calculated from genomic information and its effect on the accuracy of genomic prediction

**DOI:** 10.1371/journal.pone.0201400

**Published:** 2018-07-31

**Authors:** Suo-Yu Zhang, Babatunde Shittu Olasege, Deng-Ying Liu, Qi-Shan Wang, Yu-Chun Pan, Pei-Pei Ma

**Affiliations:** 1 Department of Animal Science, School of Agriculture and Biology, Shanghai Jiao Tong University, Shanghai, PR China; 2 Shanghai Key Laboratory of Veterinary Biotechnology, Shanghai, PR China; National Cheng Kung University, TAIWAN

## Abstract

The magnitude of connectedness among management units (e.g., flocks and herds) gives a reliable estimate of genetic evaluation across these units. Traditionally, pedigree-based methods have been used to evaluate the genetic connectedness in China. However, these methods have not been able to yield a substantial outcome due to the lack of accuracy and integrity of pedigree data. Therefore, it is necessary to ascertain genetic connectedness using genomic information (*i*.*e*., genome-based genetic connectedness). Moreover, the effects of various levels of genome-based genetic connectedness on the accuracy of genomic prediction still remain poorly understood. A simulation study was performed to evaluate the genome-based genetic connectedness across herds by applying prediction error variance of difference (PEVD), coefficient of determination (CD) and prediction error correlation (*r*). Genomic estimated breeding values (GEBV) were predicted using a GBLUP model from a single and joint reference population. Overall, a continued increase in CD and r with a corresponding decrease in PEVD was observed as the number of common sires varies from 0 to 19 regardless of heritability levels, indicating increasing genetic connectedness between herds. Higher heritability tends to obtain stronger genetic connectedness. Compared to pedigree information, genomic relatedness inferred from genomic information increased the estimates of genetic connectedness across herds. Genomic prediction using the joint versus single reference population increased the accuracy of genomic prediction by 25% and lower heritability benefited more. Moreover, the largest benefits were observed as the number of common sires equals 0, and the gain of accuracy decreased as the number of common sires increased. We confirmed that genome-based genetic connectedness enhanced the estimates of genetic connectedness across management units. Additionally, using the combined reference population substantially increased accuracy of genomic prediction. However, care should be taken when combining reference data for closely related populations, which may give less reliable prediction results.

## Introduction

The reliability of genetic evaluations across management units (e.g., flocks and herds) depends on the magnitude of connectedness among these units. Comparisons of estimated breeding values (EBVs) tend to be biased when poor connectedness exists across units[1]. The lower the connectedness across units, the larger the bias and thus, decreasing the accuracy of comparison of EBVs across units. It was reported that few highly selected sires from dairy cattle populations generally have strong genetic links owing to the wide use of artificial insemination (AI)[2]. However, it is not the case in sheep, beef cattle or pig populations where AI is less used, leading to poor or no genetic connectedness across management units. Therefore, it is necessary to estimate connectedness among management in these species units before conducting genetic evaluation across these units.

Traditionally, genetic connectedness can be calculated through pedigree-based method [1–4]. However, the pedigree information used in China cannot guarantee its integrity and accuracy, which in turn may lead to lower or unreasonable estimates of genetic connectedness across pig nucleus farms in China[3, 5, 6]. The lack of extensive and reliable pedigree information is a general problem in developing countries[7], particularly in China, where the source of the pigs are extremely complex (e.g., introduced pigs from Denmark, the United States, Canada and France). Therefore, actual genetic connectedness among Chinese pig farms might not be totally reflected by pedigree information due to the inconsistence pedigree recording system between China and the foreign countries [2]. Moreover, Yu *et al*. [8] confirmed that genomic relatedness inferred from genomic information (*i*.*e*., single nucleotide polymorphisms, SNPs) increased the estimates of genetic connectedness across different management units, compared with pedigree information. Therefore, with regards to the above opinions, it is possible to ascertain genetic connectedness through genomic information, and this can be perceived as a plausible solution to get more accurate estimates of genetic connectedness across pig farms in China, as well as enhance the genetic improvement of Chinese pigs.

Recently, connectedness statistics have been used in genomic selection[9] for the sake of optimizing the design of reference population[10, 11]. However, it is important to investigate the effect of enhanced genetic connectedness estimated by genomic relatedness on the accuracy of genomic prediction, as noted by Yu *et al*.[8].

In this study, we simulated two populations which were applied to mimic existing China pig populations with the aim to measure genetic connectedness across management units (*i*.*e*., populations) by using genomic information and also investigate the effect of various levels of genetic connectedness across herds on the accuracy of genomic prediction.

## Materials and methods

### Simulation

A simulation scheme presented by E.C. Akanno[12] was used to mimic pig breeding programs in developing countries, which was adopted in our study to mimic the situation in China. The software QMSim[13] was used to simulate the genomic data and the whole simulation process was repeated nine times. QMSim software was designed to simulate a broad range of genetic architectures and population structures in livestock. Large-scale genotyping datasets and multiple livestock pedigrees can be reliably simulated. Simulation of populations was carried out in two steps: 1) to create historical population for establishing mutation-drift equilibrium, and 2) to simulate recent population, which can be very complex. A wide range of parameters (e.g., number of chromosomes, QTL and markers, crossover interference and location of QTL and markers) are available in order to simulate appropriate genome. This simulator is efficient in time and memory[13].

#### Population structure

The populations were generated in three steps. In the first step, 1000 generations with a gradual decrease in population size from 5000 to 1050 were simulated, and then the population size was further decreased from 1050 to 200 in the following 1000 generations for the purpose of creating initial linkage disequilibrium (LD) and establishing mutation-drift equilibrium in historical population (HP).

In the second step, an expanded population (EP) was simulated by randomly choosing the 100 founder males and 100 founder females from the last generation of HP. Here, in order to expand the population, six generations was simulated assuming 10 offspring per dam under random mating.

**Fig 1:**
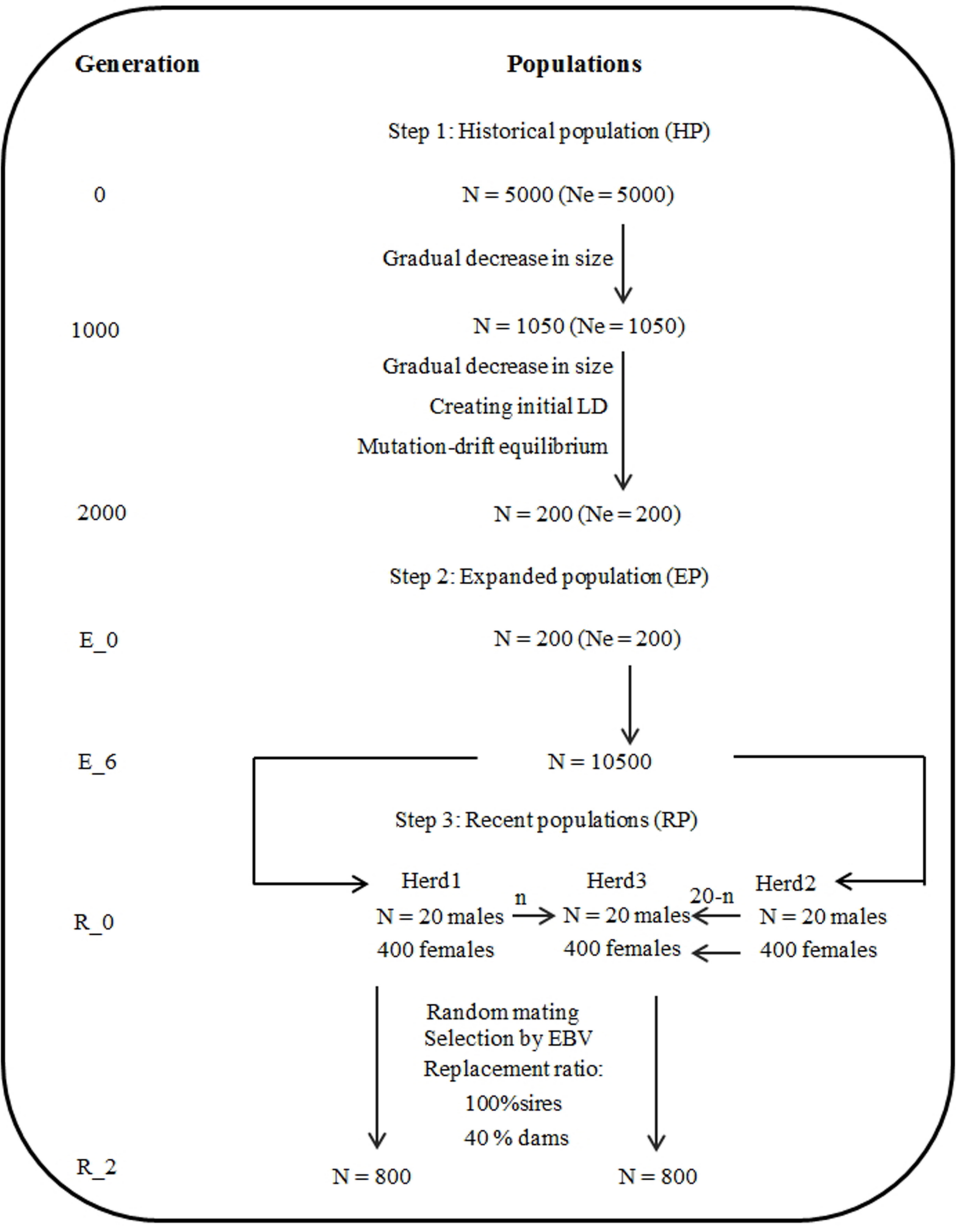
A sketch map of simulation process. Note: Ne: effective population size; LD: linkage disequilibrium.

In the third step, three recent populations (RP) (*i*.*e*., Herd1, Herd2 and Herd3) were simulated, and each of them with the population size of 20 founder males and 400 founder females from the last generation of EP. The size defined above represented the median group size for pig nucleus farms in China. The Herd1 population was composed of the top 20 males and top 400 females on the basis of their own phenotypic values from the EP. In order to make Herd1 have no connection with Herd2, Herd2 was simulated by selecting the last 20 males and the last 400 females from the EP. It is well recognized that genetic connectedness among China pig herds was generally established through using of common sires (*i*.*e*., sires with progeny in multiple herds or sires born in one herd with progeny in another herd) or through transferring of seedstock from one herd to another[3]. Therefore, to mimick the genetic connectedness created by common sires, 400 founder females of Herd3 were all from the first generation of Herd2, while the 20 founder males of Herd3 came from Herd1 and Herd2. It is assumed that the number of males defined as common sires from the founder males of Herd1 is n (0 ≤ n ≤ 19), then the remaining males from Herd2 is 20—n. Increasing n increased the genetic connectedness between Herd1 and Herd3. Moreover, the RP parameters used in this study mimicked more closely to a real Chinese pig production system with selection for high values of EBV and culling for low values of EBV with a replacement rate of 100% for sires and 40% for dams. Best linear unbiased prediction (BLUP) method was used to estimate the breeding value by using the Henderson’s mixed model theory[14] for an animal model. In this study, three traits corresponding number born alive, average daily gain and backfat were mimicked, whose heritability and phenotypic variance were obtained from a previous study carried out by Akanno E *et al*. [15]. Considering the computing time and memory requirements, only two generations of each RP were simulated. Herd1 and Herd3 both had 2020 individuals, which were made up of 420 founders and 800 progenies each from the first and second generation. Details of the parameters used to generate genomic data are given in Table 1, while the simulation steps are described in Fig 1.

**Table 1:** Parameters of the simulation process.

Population structure	Parameters
**Step1: Historical population (HP)**	
Number of generations (size)–phase 1	1000 (1050)
Number of generations (size)–phase 2	1000 (200)
**Step2: Expanded population (EP)**	
Number of males from HP	100
Number of females from HP	100
Number of generations	6
Number of offspring per dam	10
**Step3: Recent populations (RP)**	
Number of males from EP	20
Number of females from EP	400
Number of offspring per dam	2
Ratio of male	0.5
Number of generations	2
Replacement ratio for males	100%
Replacement ratio for females	40%
Selection /culling	EBV
Breeding value estimation method	BLUP
**Traits**	
Number born alive	h^2^ = 0.08, $\sigma_{p}^{2}$ = 7.73
Average daily gain, g/d	h^2^ = 0.28, $\sigma_{p}^{2}$ = 10361.20
Backfat, mm	h^2^ = 0.63, $\sigma_{p}^{2}$ = 20.88
**Genome**	
Number of chromosomes	18
Genome length per chromosome	100 cM
Number of markers per chromosome	3300
Number of QTL per chromosome	25
Minor allele frequency (MAF)	≥ 0.05
Mutation rate of marker locus	2.5 × 10^-3^
Mutation rate of QTL locus	2.5 × 10^-5^

EBV: estimated breeding value; BLUP: best linear unbiased prediction; h^2^: heritability; $\sigma_{p}^{2}$: phenotypic variance; QTL: quantitative trait loci.

#### Genome

The genome parameters were consistent with a previous study conducted by [16]. In this study, in order to create more realistic pig genome size, each chromosome was simulated to acquire an average length of 100 cM[17]. The marker density represented approximately 60 K SNP chip currently available[18]. The parameters shown in Table 1 were used to simulate the genome.

### Genetic connectedness criteria

We used prediction error variance (PEV) of differences (PEVD), generalized coefficient of determination (CD) and prediction error correlation (*r*) defined below to investigate genetic connectedness between Herd1 and Herd3. Here, the PEV were obtained from the Henderson’s mixed model equation (MME) [14] and the PEV of *i*th individual is given by

$${\mathrm{PEV}}_{i}{=D}_{22}^{\mathrm{ii}}\sigma_{\varepsilon }^{2}$$

Where $D_{22}^{\mathrm{ii}}$ is the *i*th diagonal element of **D**_22_ coefficient matrix which is defined as the inverse of the MME coefficient matrix (**D**) corresponding to genetic values. $\sigma_{\varepsilon }^{2}$ is the residual variance. A detailed description of the genetic connectedness criteria was provided by Yu et al [8].

PEVD, the average PEV of all pairwise EBV differences between the individuals across management units[2], which is calculated as

$$\mathrm{PEVD}\left({û}_{i}{‑û}_{j}\right)=\mathrm{PEV}\left({û}_{i}\right)+\mathrm{PEV}\left({û}_{j}\right)‑2\mathrm{PEC}\left({û}_{i}{,û}_{j}\right)=\left(D_{22}^{\mathrm{ii}}{+D}_{22}^{\mathrm{jj}}{‑2D}_{22}^{\mathrm{ij}}\right)\sigma_{\varepsilon }^{2}$$

Where ${û}_{i}$ and ${û}_{j}$ represent genetic value for individual i and individual j, respectively. PEC_ij_ indicates the prediction error covariance (PEC) defined by the off-diagonal element of the PEV matrix. The PEVD is used as a criterion to measure the genetic connectedness because poor connectedness among individuals will have higher prediction error than strong connectedness. In this study, a scaled PEVD was used for further analysis based on Kuehn’s suggestion[19]. Smaller PEVD indicated stronger connectedness.

CD, generalized coefficient of determination[20], is calculated as follows

$${\mathrm{CD}}_{\mathrm{ij}}=1‑\lambda \frac{D_{22}^{\mathrm{ii}}{+D}_{22}^{\mathrm{jj}}{‑2D}_{22}^{\mathrm{ij}}}{R_{\mathrm{ii}}{+R}_{\mathrm{jj}}{‑2R}_{\mathrm{ij}}}$$

Where **λ**, $D_{22}^{\mathrm{ii}}$, $D_{22}^{\mathrm{jj}}$, $D_{22}^{\mathrm{ij}}$ are the same values defined above, and **R** is a relationship matrix which measures the relationship between individuals (defined below). This statistic ranging from 0 to 1 with larger values represented stronger connectedness.

And the *r* between genetic values of individuals from different management units is derived as[4].

$$r_{\mathrm{ij}}=\frac{\mathrm{PEC}\;\left({û}_{i}{,û}_{j}\right)}{\sqrt{\mathrm{PEV}\;\left({û}_{i}\right)\;\mathrm{PEV}\;\left({û}_{j}\right)}}$$

Similar to CD, the statistic *r* also ranged from 0 to 1 and larger *r* indicated stronger connectedness across management groups.

### Relationship matrix

Connectedness is determined in BLUP framework using the genetic relationship matrix. The information about the covariance structures among individuals is required to estimate the relatedness of the three genetic connectedness criteria stated above[8]. In this study, four relationship matrices (**R**) measuring the relationship among individuals are the same as previous study provided by Yu et al [8] and are defined below.

Firstly, **R** = **A**^**PED**^, the usual numerator relationship matrix. When genetic evaluation is under an animal model, connectedness occurs due to **A**^**PED**^[2]. The **A**^**PED**^ is directly calculated from the known pedigree and denotes the probability of inheritance of alleles from a common ancestor indicating that they are identical by descent (IBD). The off-diagonal elements are twice coefficients of kinship and are equivalent to the numerators of Wright’s correlation coefficients[21].

Secondly, **R** = **G**^**BASE**^, basic genomic relationship matrix **G**^**BASE**^ was constructed according to the method (method 1) described by VanRaden[22], *i*.*e*., $G^{\mathrm{BASE}}=\frac{{\mathrm{MM}}^{'}}{\sum {2p}_{i}{(1‑p}_{i})}$, where elements in column i of M are **0-2p**_**i**_, **1-2p**_**i**_ and **2-2p**_**i**_ for genotypes **A**_**1**_**A**_**1**_, **A**_**1**_**A**_**2**_ and **A**_**2**_**A**_**2**_, respectively, and **p**_**i**_ is the allele frequency of **A**_**2**_ at locus i, calculated from the available marker data, as negative values generated in this scenario, **R = G**^**0.5**^ (*i*.*e*., the third matrix), which supposes the MAF in the base population is unknown and 0.5 is used for all **p**_**i**_. The **G**^**0.5**^ constructed in this way does not create any negative values for simulated data.

Fourthly, when comparing marker-based with pedigree-based relationship matrices, scaling of genomic relationship matrices is needed for interpretation of genetic connectedness criteria. A reasonable rescaling may be achieved by using genomic elements that ranged between 0 and 2, which are the minimum and maximum values of **A**^**PED**^, respectively. Therefore, to render **G**^**BASE**^ on the same scale as **A**^**PED**^, a scaled **G**^**BASE**^ matrix (**G**^**S**^) was created and the scaled genomic relationship between *i*th and *j*th individual was given by

$${\mathrm{Gs}}_{\mathrm{ij}}=\frac{{(\mathrm{Gs}}_{\max}{‑\mathrm{Gs}}_{\min}{)(G}_{\mathrm{ij}}{‑G}_{\min})}{G_{\max}{‑G}_{\min}}{+\mathrm{Gs}}_{\min}$$

Where **Gs**_**ij**_ is a scaled element of the **G**^**BASE**^ and **G**_**ij**_ is a typical element of **G**^**BASE**^. **Gs**_**max**_ = 2 and **Gs**_**min**_ = 0 are the maximum and minimum values elements that the scaled matrix is allowed to take, respectively, while **G**_**max**_ and **G**_**min**_ are the maximum and minimum element of the **G**^**BASE**^. In this case, **G**^**S**^ does not create any negative values.

Finally, in order to simulate a more realistic scenario where not all the individuals were genotyped in the population, the **H** matrix (*i*.*e*., relationship matrix with pedigree and genomic information) was given by [23–25]

$$Η=\left[\begin{array}{cc} G_{\omega } & G_{\omega }A_{11}^{‑1}A_{12}\\ A_{12}^{T}A_{11}^{‑1}G_{\omega } & A_{22}{+A}_{12}^{T}A_{11}^{‑1}{(G}_{\omega }{‑A}_{11}{)A}_{11}^{‑1}A_{12} \end{array}\right]$$

where the **A**_**11**_, **A**_**22**_ and **A**_**12**_ are submatrices of **A** matrix representing relationships among genetyped, among non-genotyped, and between genotyped and non-genotyped individuals respectively, and the superscript T represents the transpose of a matrix. The **G**_**ω**_ matrix indicates relationship of genotyped individuals and defined as

$$G_{\omega }{=(1‑\omega )G+\omega A}_{11}$$

where the **ω** represents the fraction of the genetic variance not captured by markers, and **G = G**^**BASE**^, **G**^**0.5**^ and **G**^**S**^ defined above. In this study, we assumed that individuals at generation 0–1 (N = 2440) as non-genotyped individuals while individuals from generation 2 (N = 1600) were genotyped. This simulated a real scenario, where individuals from more recent generation were likely to be genotyped with a relatively small sample size compared with individuals from earlier generations.

### Population structures of the simulated populations

Principle component analysis (PCA) was used to investigate the population structure of Herd1 and Herd3. PCA was performed using PLINK software[26] and the PC plots were drawn by the ggplot2 package[27].

### Prediction of genomic breeding values

In order to investigate the impact of various genetic connectedness inferred from genomic information on the accuracy of genomic prediction, the genomic breeding values were predicted using GBLUP, with different genomic matrices (**G**^**BASE**^, **G**^**0.5**^ and **G**^**S**^) defined above. In addition, we also examined the predictive ability of other two relationship matrices (*i*.*e*., **A**^**PED**^ and **H**) to better understanding the possible effects of genomic connectedness on genomic prediction. The model was the same as the GBLUP model shown below but genomic relationship matrices were replaced by **A**^**PED**^ and **H** when predicting the (G) EBV.

The basic GBLUP model [22, 28] was defined as:

y=1μ+Ζg+ε

Where **y** is simulation phenotypes, **μ** is the population mean, **g** is the vector of breeding values, **ε** is the vector of residuals, **Z** is an appropriate design matrix. Assuming that $g∼N\left(0,G\sigma_{g}^{2}\right)$ and $\varepsilon ∼N\left(0,I\sigma_{\varepsilon }^{2}\right)$, where **G** is the genomic relationship matrix. $\sigma_{g}^{2}$ is the additive genetic variance, **I** is the identity matrix and $\sigma_{\varepsilon }^{2}$ is the residual variance.

### Reference and validation data

The Herd1 data were divided into reference data and validation data by generation. The reference population was made up of a total of 1220 individuals comprising of 420 founders and 800 progenies from the first generation. The validation population comprised of 800 individuals from the second generation. To avoid inflation of the accuracy of genomic prediction, 1220 individuals from the founders and the first generation of Herd3 were included in a joint reference population. The accuracy of genomic prediction was estimated as the correlation between predicted genomic estimated breeding values (GEBV) and the true breeding values of the animals in the validation set.

## Results

### Genetic connectedness criteria

**Fig 2:**
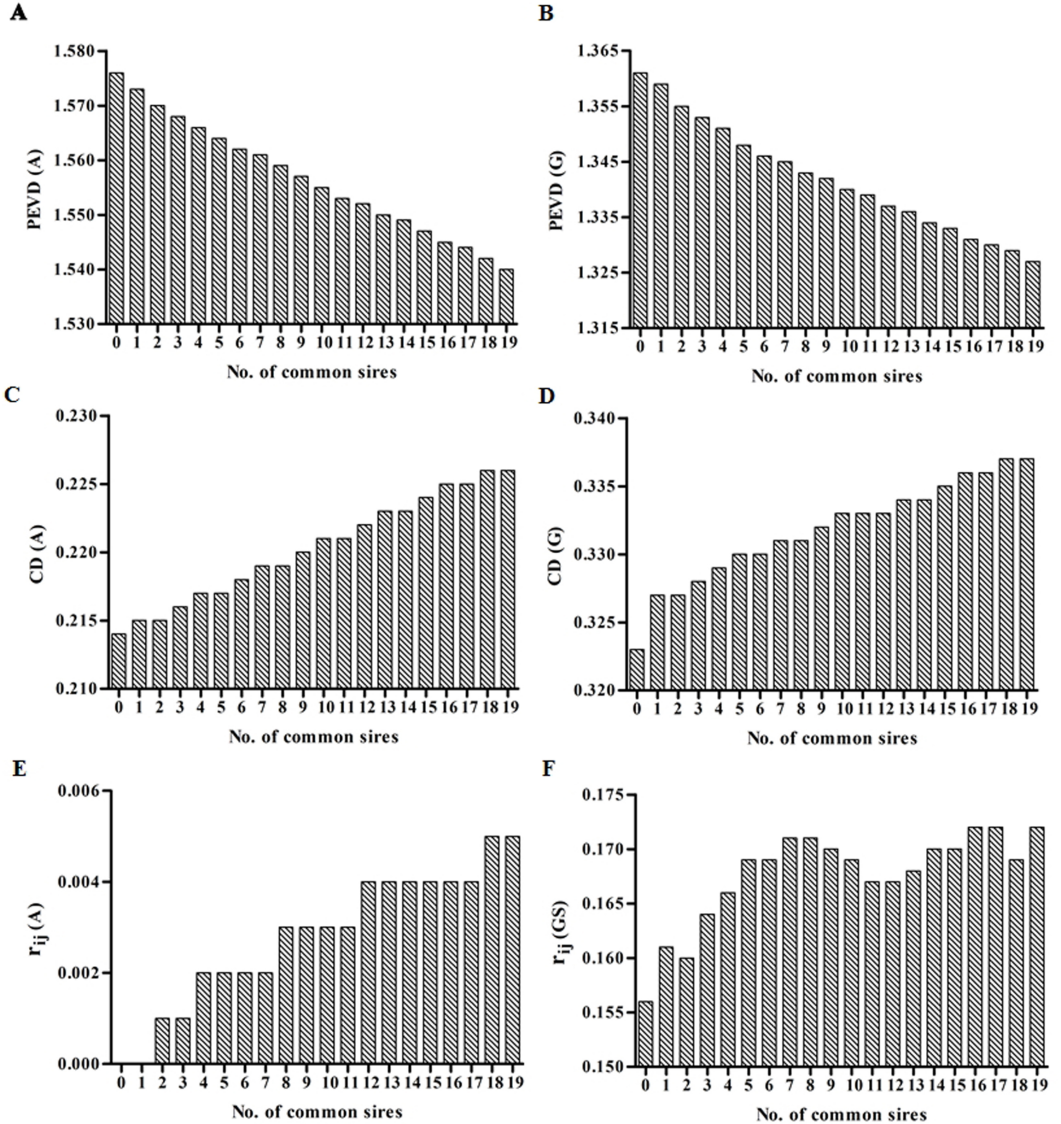
The estimates of PEVD, CD and *r*_*ij*_ at heritability = 0.08. Left column: **A**^**PED**^. Right column: **G**^**BASE**^. For *r*_*ij*_, the **G**^**BASE**^ was replaced by **G**^**S**^.

**Fig 3:**
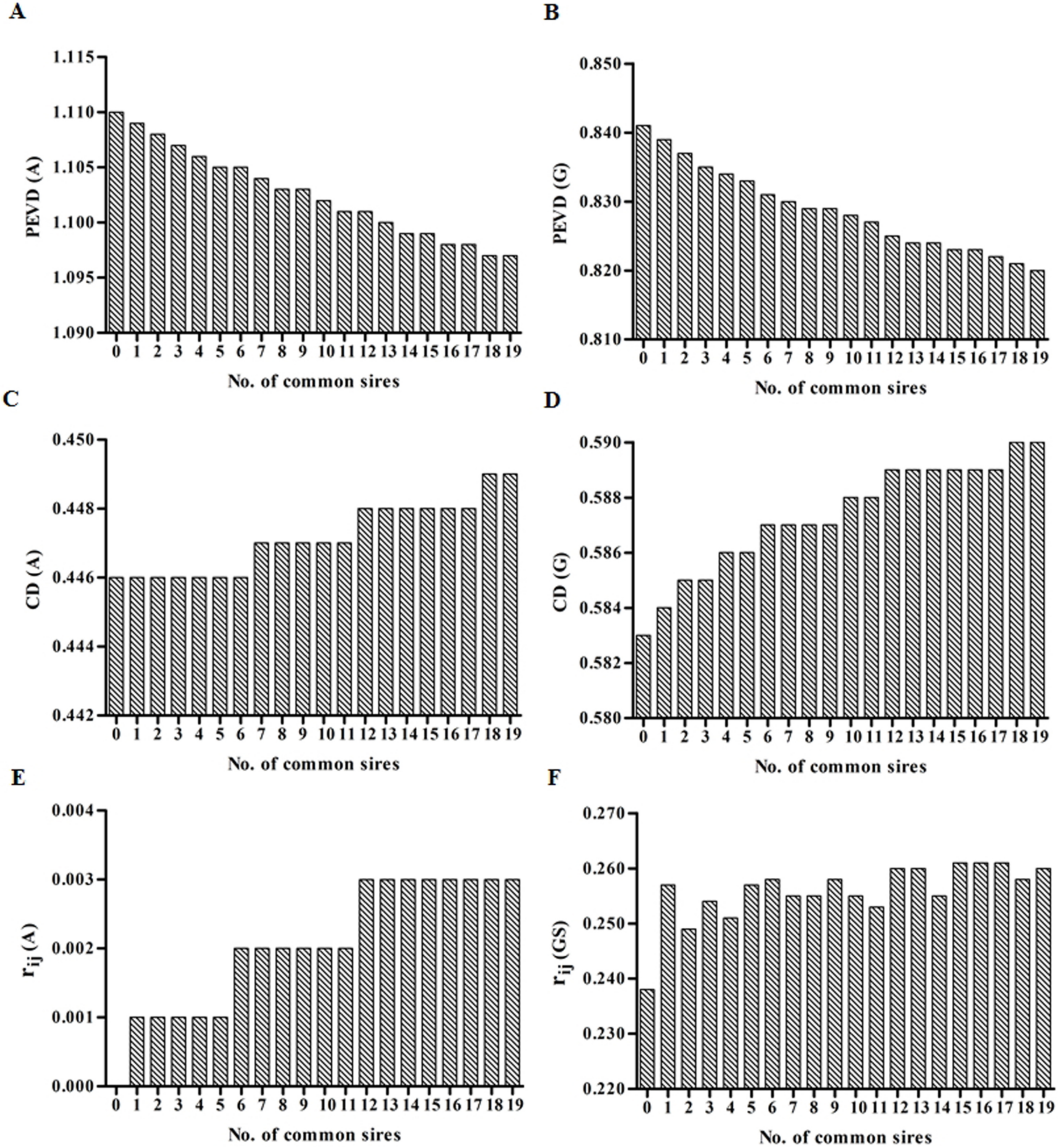
The estimates of PEVD, CD and *r*_*ij*_ at heritability = 0.28. Left column: **A**^**PED**^. Right column: **G**^**BASE**^. For *r*_*ij*_, the **G**^**BASE**^ was replaced by **G**^**S**^.

**Fig 4:**
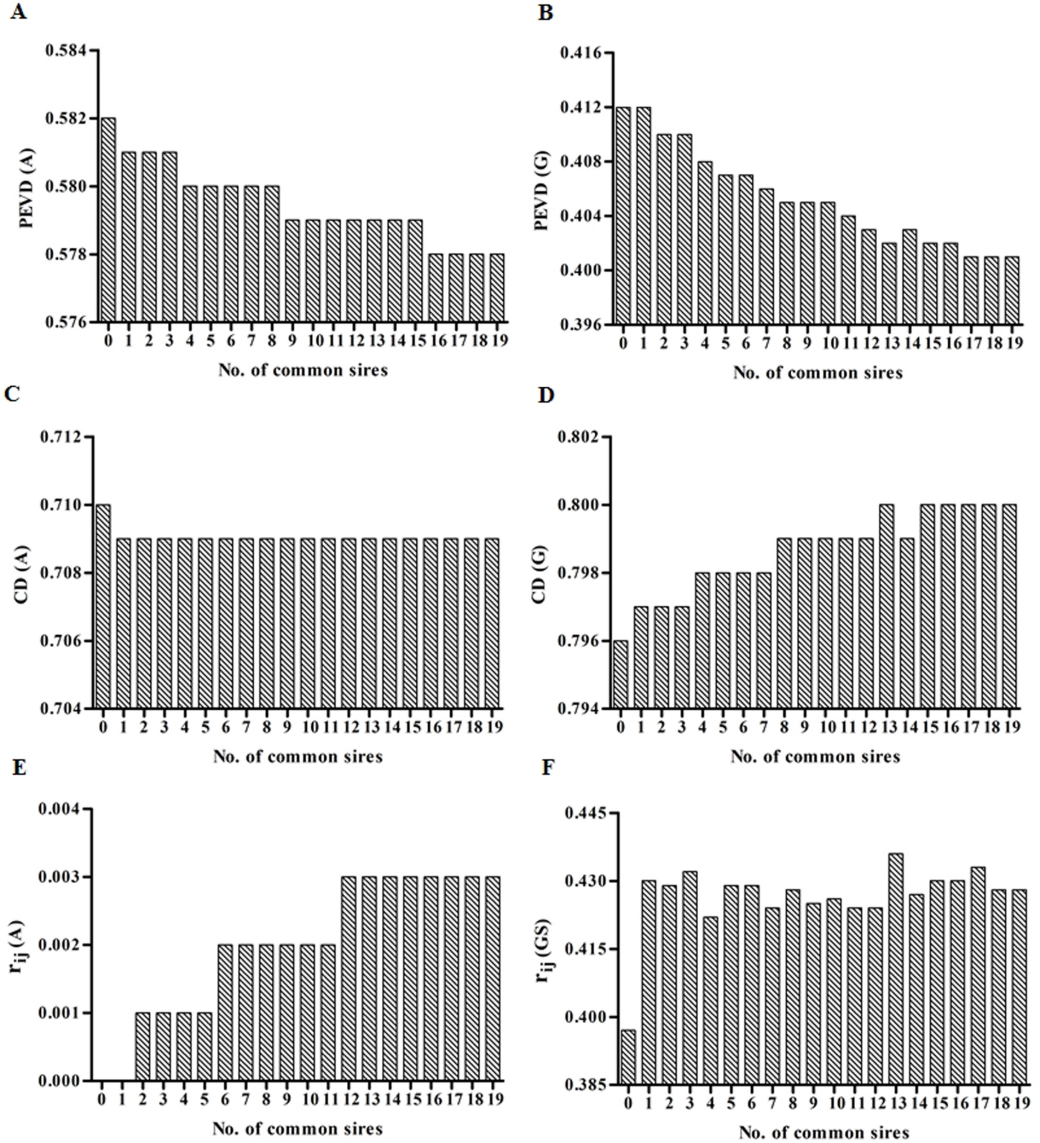
The estimates of PEVD, CD and *r*_*ij*_ at heritability = 0.63. Left column: **A**^**PED**^. Right column: **G**^**BASE**^. For *r*_*ij*_, the **G**^**BASE**^ was replaced by **G**^**S**^.

Genetic connectedness criteria between Herd1 and Herd3 for varied number of common sires with heritability of 0.08, 0.28 and 0.63 were presented in Fig 2, Fig 3 and Fig 4, respectively. Similar results among PEVD, CD and *r*_*ij*_ were observed.

Firstly, the increasing number of common sires (ranged from 0 to 19) increased the estimates of CD and *r*_*ij*_ but decreased PEVD in each heritability level when **A**^**PED**^ was used, indicating an increasing level of connectedness across herds. However, owing to the very high existing values of CD (CD for **A**^**PED**^) with almost no change at the heritability of 0.63 (0.709–0.71) among common sires, hence, any further increase in CD might be difficult. A similar trend was also observed for **G**^**BASE**^. As the number of common sires increased, the CD and *r*_*ij*_ increased with a decrease in PEVD indicating stronger genetic links between herds. Note that **G**^**BASE**^ *r*_*ij*_ criteria behave erratically with negative values, making them difficult to interpret. Thus **G**^**S**^ instead of **G**^**BASE**^ was used in comparison with **A**^**PED**^. As shown in Fig 2, Fig 3, Fig 4 and Supporting Information (S1 Table), for **G**^**S**^, three criteria occasionally fluctuated with increasing number of common sires, particularly for lower heritability levels. However, the general trend for the level of connectedness increased with the increasing number of common sires.

Secondly, as heritability increased, the levels of connectedness all increased regardless of genetic connectedness criteria, except for *r*_*ij*_ in **A**^**PED**^ in which the estimates for different heritability levels appeared similar (ranged from 0.001 to 0.005).

Finally, the estimates of **G**^**BASE**^, **G**^**0.5**^, and **G**^**S**^ for different heritability levels were all higher than that of **A**^**PED**^ (as seen in S1 Table). As expected, the *r*_*ij*_ estimates were all 0 in relation to **A**^**PED**^ when number of common sires equal to 0 regardless of heritability levels. This is because PEC among completely disconnected datasets all equals to 0.

We also simulated a more realistic scenario that only individuals in earlier generations were genotyped in the simulated dataset. In this case, the genomic matrices (*i*.*e*., **G**^**BASE**^, **G**^**0.5**^, and **G**^**S**^) were combined with the **A**^**PED**^ creating **H** matrices. As shown in Supporting Information (S3 Table), estimates obtained from **H** matrix lie somewhere between the estimates observed when using the **A**^**PED**^, **G**^**BASE**^, **G**^**0.5**^, and **G**^**S**^. This is reasonable due to the fact that the **H** matrix was constructed based on a combination of **A**^**PED**^ and the genomic matrices (*i*.*e*., **G**^**BASE**^, **G**^**0.5**^, and **G**^**S**^). Very little differences in the estimates were observed when **A**^**PED**^ was combined with **G**^**BASE**^, **G**^**0.5**^ and **G**^**S**^ and thus only results for **G**^**BASE**^ were shown (S3 Table).

### PCA of the simulated populations

**Fig 5:**
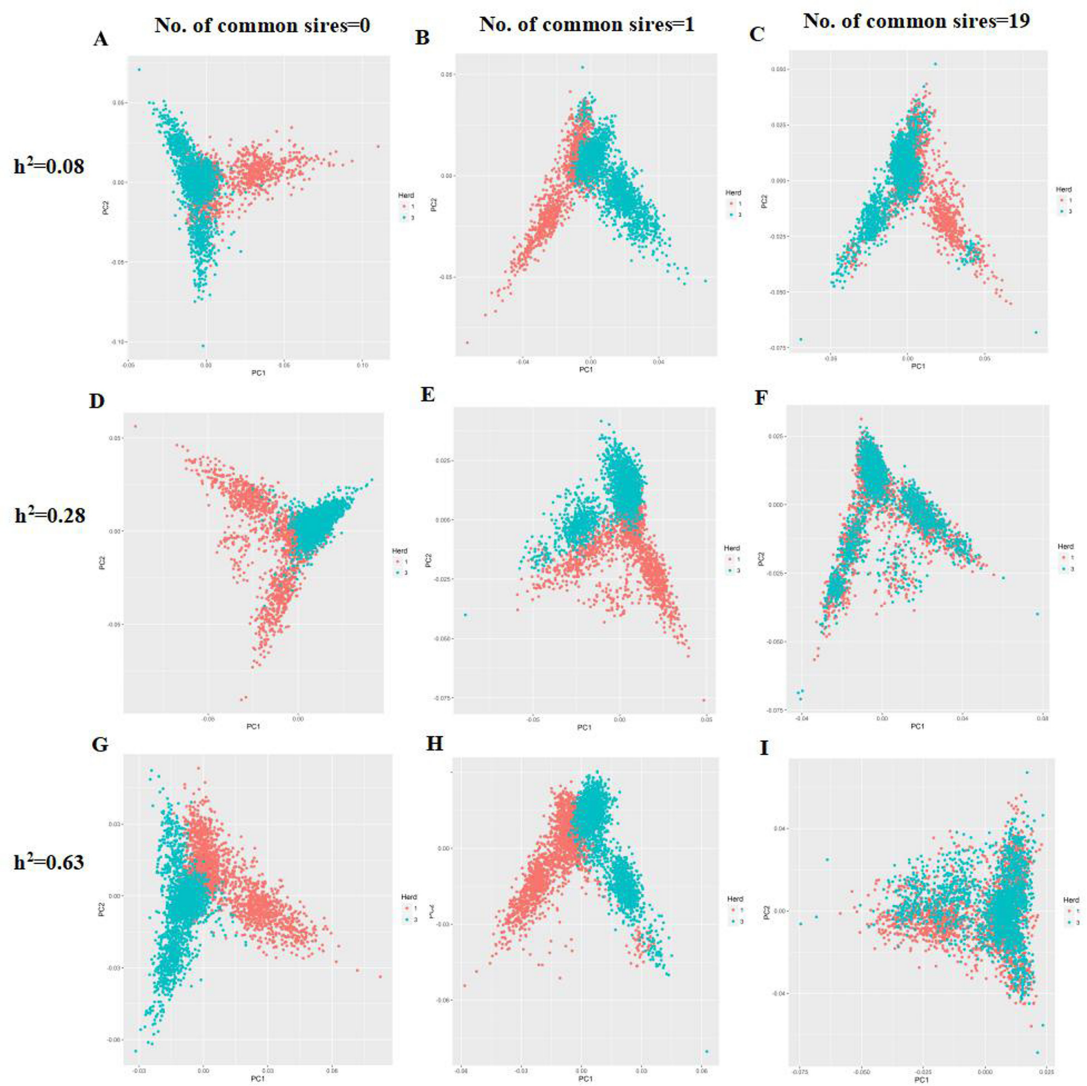
Principal component analysis plots for the simulated populations. PC1: Principal component 1. PC2: Principal component 2. Red: Herd1. Blue:Herd3.

For the PCA, the first two principal components did not clearly separated all individuals from Herd1 and Herd3 into their respective groups when the number of common sires equal to 0 regardless of heritability levels (Fig 5A, Fig 5D and Fig 5G). As the number of common sires increased, all individuals tend to cluster together as expected, especially for number of common sires equal to 19 (Fig 5C, Fig 5F and Fig 5I).

### Genomic prediction

**Fig 6:**
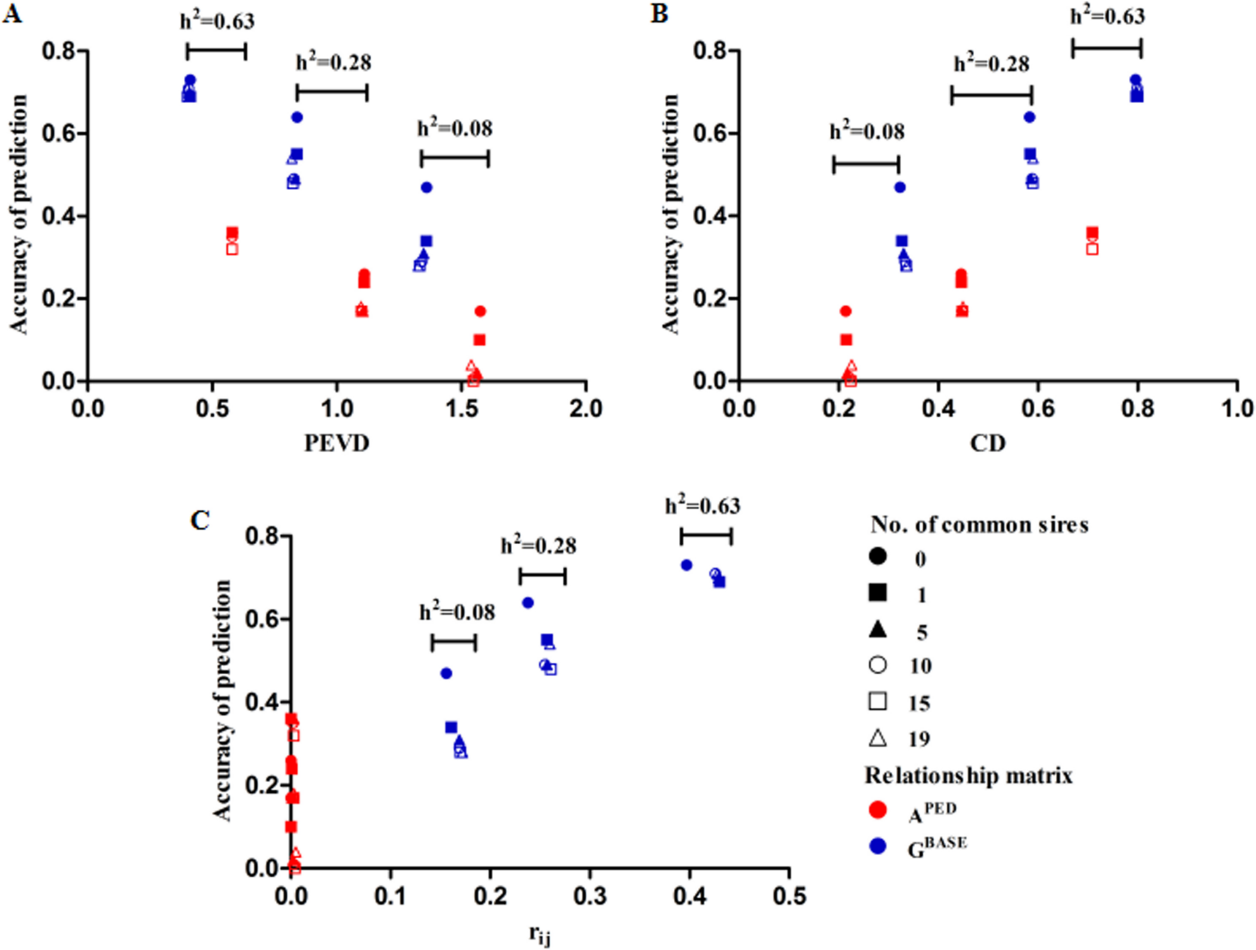
The relationship between genetic connectedness criteria and accuracy of prediction. For *r*_*ij*_, the **G**^**BASE**^ was replaced by **G**^**S**^ and the estimates of **A**^**PED**^ did not clearly distinguish the *r*_*ij*_ values at different heritability levels due to relatively small values (ranged from 0 to 0.005).

Accuracy of genomic prediction using Herd1 reference population or joint reference populations (Herd1 + Herd3) for specified scenarios was presented in Table 2. Compared to genomic prediction using Herd1 reference population alone, the accuracy of genomic prediction using joint reference population increased by 25% averaged over common sires, heritability levels and genomic relationship matrices (the detailed results are provided as Supporting Information (S2 Table)). Lower heritability benefited more. Moreover, it is worthy to note that the largest benefits were observed when the number of common sires equal to 0, and the gain of accuracy becomes smaller as the number of common sires increased. Additionally, the accuracy of genomic prediction using **G**^**BASE**^ was consistent with **G**^**0.5**^ and **G**^**S**^ in each heritability level regardless of the scenarios. Furthermore, for **A**^**PED**^ and **G**^**BASE**^, as the number of common sires increased, the accuracy of prediction generally decreased with increasing the CD and *r*_*ij*_ and decreasing PEVD regardless of heritability levels (Fig 6, the detailed results are provided as Supporting Information (S2 Table)). The highest accuracy was observed when the number of common sires equal to 0, as reflected by the lowest CD and *r*_*ij*_ values and highest PEVD estimates.

**Table 2:** Accuracies of (G)EBV in the validation population when using the Herd1 or the joint reference population.

No. of common sires^1^	Heritability	Relationship^2^ matrix	Accuracy of prediction^5^		
			Herd1 reference^3^	Joint reference^4^	Increase
0					
	0.08				
		**G**^**BASE**^	0.03	0.47	0.44
		**G**^**0.5**^	0.03	0.47	0.44
		**G**^**S**^	0.03	0.47	0.44
		**A**^**PED**^	0	0.17	0.17
	0.28				
		**G**^**BASE**^	0.20	0.64	0.44
		**G**^**0.5**^	0.20	0.64	0.44
		**G**^**S**^	0.20	0.64	0.44
		**A**^**PED**^	0	0.26	0.26
	0.63				
		**G**^**BASE**^	0.55	0.73	0.18
		**G**^**0.5**^	0.55	0.73	0.18
		**G**^**S**^	0.55	0.72	0.17
		**A**^**PED**^	0.31	0.36	0.05
1					
	0.08				
		**G**^**BASE**^	0.03	0.34	0.31
		**G**^**0.5**^	0.03	0.35	0.32
		**G**^**S**^	0.03	0.33	0.30
		**A**^**PED**^	0	0.10	0.10
	0.28				
		**G**^**BASE**^	0.20	0.55	0.35
		**G**^**0.5**^	0.20	0.56	0.36
		**G**^**S**^	0.20	0.55	0.35
		**A**^**PED**^	0	0.24	0.24
	0.63				
		**G**^**BASE**^	0.55	0.69	0.14
		**G**^**0.5**^	0.55	0.70	0.15
		**G**^**S**^	0.55	0.69	0.14
		**A**^**PED**^	0.31	0.36	0.05
19					
	0.08				
		**G**^**BASE**^	0.03	0.28	0.25
		**G**^**0.5**^	0.03	0.29	0.26
		**G**^**S**^	0.03	0.28	0.25
		**A**^**PED**^	0	0.04	0.04
	0.28				
		**G**^**BASE**^	0.20	0.54	0.34
		**G**^**0.5**^	0.20	0.54	0.35
		**G**^**S**^	0.20	0.53	0.33
		**A**^**PED**^	0	0.18	0.18
	0.63				
		**G**^**BASE**^	0.55	0.71	0.16
		**G**^**0.5**^	0.55	0.71	0.16
		**G**^**S**^	0.55	0.70	0.15
		**A**^**PED**^	0.31	0.36	0.05

^1^Common sires = 0 (completely disconnected scenario between Herd1 and Herd3); common sires = 1 (connected scenario); common sires = 19 (strongly connected scenario). Increasing common sires increased the level of connectedness between herds.

^2^**A**^**PED**^ = the usual numerator relationship matrix; **G**^**BASE**^ = standard genomic relationship matrix; **G**^**0.5**^ = genomic relationship matrix assuming 0.5 minor allele frequency; **G**^**S**^ = a scaled genomic relationship matrix.

^3^Herd1 reference: reference population only consisting of individuals from Herd1.

^4^Joint reference: reference population consisting of individuals from both Herd1 and Herd3.

^5^Standard errors for accuracy of prediction ranging from approximately 0.023 to 0.121

In order to gain a better understanding of the possible effects of genomic connectedness on genomic prediction, the accuracies of the genomic predictions based on **A**^**PED**^ and **H** matrix were investigated as a comparison. Similar to the genomic matrices, **A**^**PED**^ and **H** matrix both gained (increased accuracy of genomic prediction) from using combined reference population (increased by on average 9% and 14%, respectively), with the largest gain for number of common sires equal to 0 and the gain of accuracy decreased as the number of common sires increased. In addition, relationship matrix with marker information (**G**^**BASE**^, **G**^**0.5**^, **G**^**S**^ and **H** matrix) provided higher accuracies of predictions than **A**^**PED**^ regardless of heritability levels and scenarios (*i*.*e*., varied number of common sires) (detailed information see S2 Table, S4 Table).

## Discussion

The EBVs of individuals across management units (*i*.*e*., contemporary groups or herds) are comparable due to the use of BLUP method in genetic evaluation. However, the accuracy of these comparisons depends on the extent of connectedness among these units. The lack of the integrity and accuracy of the pedigree in China pig farms may lead to several practical problems. The use of pedigree-base methods (result unpublished) revealed no genetic links among pig nucleus farms such as BJLM, AHCF and FQYC in China. But in reality, there are possibilities of genetic connectedness existing among them due to common sire and also, since they all purchased seedstock from the same company. In such case, advancement of molecular biotechnology can provide novel insights to ascertain genetic connectedness at the genomic level. Our results confirmed that genomic relatedness increased the estimates of genetic connectedness across herds compared with its counterpart (*i*.*e*., pedigree relationship). Moreover, when reference datasets were combined, the accuracy of genomic predictions, averaged over each common sire scenarios, heritability levels and genomic relationship matrices, increased by 25% compared to genomic prediction using Herd1 reference alone. The largest benefits were observed as the number of common sires equal to 0 and the gain of accuracy of genomic prediction was smaller as the number of common sire increased.

### The effect of genomic information on genetic connectedness

Pedigree-based genetic connectedness across management units has caused a great concern in the field of animal breeding. However, connectedness ascertained by genomic information was still remains poorly understood. The result from our study confirmed that genomic information enhance the estimates of genetic connectedness across the herds using PEVD, CD and *r* criteria regardless of heritability levels, and this is consistent with previous study of Yu *et al*. [8]. In 2017, Yu *et al*. proved that genomic relatedness strengthened genetic connectedness among management units by using the same genetic connectedness criteria. Given these data, the reason for the improved genetic connectedness might be due to the genomic relatedness captured Mendelian sampling which does not exist in pedigree relationship[29].

### Genetic connectedness criteria

In order to provide a better understanding of the measurements of genetic connectedness, three known criteria (*i*.*e*., PEVD, CD and *r*) were used in this study. Overall, genetic connectedness calculated by PEVD and *r* criteria increased as the growth of common sires increases, which was in accordance with previous study[8]. However, the continued growth in CD relative to the increasing number of common sires differed from those reported by Yu et al [8]. Laloë D noted that CD is dependent on PEV and genetic variability[30]. The possible reason for the differences observed in the former and latter results might be due to the genetic variability in two generations simulated in the latter study which remained constant throughout the period of the study. In this case, a decrease in PEV corresponds to an increase in CD, which was confirmed in the present study. On the contrary, we speculated that the genetic variability tend to change because relatively intensive selection might have occurred in the previous studies.

In addition to PEVD, CD, and r, Mathur *et al*[31] proposed a connectedness statistics (the connectedness rating (CR) ranged from 0 to 1) to measure connectedness as the correlation between the estimates of the herd effects. We recalculate CR using three relationship matrices defined above, the CR statistics behave erratically in all scenarios (*e*.*g*., covariance of herd effects appeared negative values, leading negative values of CR) (result unpublished), making them difficult to interpret. The reason could be the negative values exist in the G matrix. How to apply this method in calculating genomic connectedness needs to be investigated in the future.

### Genomic prediction

The construction of a large reference population for genomic prediction is difficult for numerically small breeds and traits that are difficult to measure. Particularly in China, the reference population size for pigs is normally smaller than other livestock species and this strongly inhibits the enhancement of genomic prediction accuracy for pigs. So far, the most straightforward method to increase the reliability is to combine reference data from different populations of the same breeds or different breeds, or by using robust methods (*e*.*g*., single step method).

In this study, Herd1 and Herd3 were both from the same historical population. In such cases, they were analogous to simulate two subpopulations (*e*.*g*., two lines in pig industry) from the whole population. Thus, we tended to combine reference data from the same populations (*e*.*g*., the same breed). By combining reference data, the accuracy of genomic prediction increased by 25% compared to genomic prediction using Herd1 reference data alone (S2 Table). This accuracy was determined by estimating the average of each common sire scenarios, such as average of different heritability levels and three genomic relationship matrices. The increase in accuracy of genomic prediction in our study was in accordance with earlier reports, for instance, Yorkshire populations in China[32], Holstein Friesian in North American[33, 34], in EuroGenomics [35] and in China Holstein Friesian population[36].

The accuracy of predictions based on **A**^**PED**^ matrix were lower than that of relationship matrices with marker information (*i*.*e*., **G**^**BASE**^, **G**^**0.5**^, **G**^**S**^ and **H** matrix), which was in agreement with previous studies [37]. Moreover, the prediction accuracy of **H** matrix was generally lower than that of genomic matrices (*i*.*e*., **G**^**BASE**^, **G**^**0.5**^, and **G**^**S**^) across all scenarios and heritability levels, this is largely due to the fact that only a subset of individuals (N = 1600) were assumed to be genotyped, whereas, all individuals (N = 4040) from three generations assumed to be genotyped were used to estimate GEBV based on three genomic matrices. Based on the results of accuracy of **H** matrix, it has become increasingly apparent that single step method [23, 24] performed better than traditional BLUP method on **A**^**PED**^ even when the genotyped sample size was relatively small. This is especially important in the current China pig populations, where genomic selection is still in an early stage with limited genotyped individuals. Several earlier studies have shown that the improved genomic prediction due to combined reference population is mainly about the increased relatedness between the reference and validation populations [35]. Interestingly, as shown in S2 Table, combining two completely disconnected herds (*i*.*e*., number of common sires = 0) achieved the highest accuracy. The reasons may be attributed to the relationship between individuals from Herd1 and Herd3 which exist through genomic information if traced back far enough[38], this was confirmed by PCA plots where individuals from Herd1 and Herd3 were not clearly separated by the first two principal components when the number of common sires equal to 0 (Fig 5A, Fig 5D and Fig 5G) Therefore, the simulated data in our study is more similar to the scenario in two separate lines in one farm rather than two different herds. We found that increasing number of common sires decreased the gain of accuracies for joint reference population. It is speculated that the reason is largely due to the increasing genetic links in relation to number of common sires within reference population. Previous simulation study[39] showed that average reliabilities increased when average relationship within the reference population decreased. Moreover, Herd1 and Herd3 both from the same historical population (Ne = 200) and Ne is expected to remain constant due to their limited selection (generation = 2). In such cases, increased genetic connectedness within population may give less reliable prediction ability.

An extreme case of strong connectedness scenario was simulated to investigate its effect on the accuracy of genomic prediction. In this case, as the number of common sires across herds equal to 19 (the founder sires = 20), the individuals in generation 1 of Herd1 and Herd3 were all nearly half-sibs. Additionally, a value of 0.790 inferred from **A**^**PED**^ and 0.800 estimated by **G**^**BASE**^ both by using CD (in the range of 0 to 1) at heritability of 0.68 confirmed the strong genetic links across herds. It is pleasing to infer that, the accuracies for this extreme case in relation to strong genetic connectedness within reference data were still higher than that of using Herd1 reference data only. Consequently, the results indicated that the benefits of using the combined reference data may to some extent decrease by increasing the level of genetic connectedness within reference data. However, this is may not counteract the overall benefits of combining datasets.

### Future direction

In this study, we focused on two simulated subpopulations (*e*.*g*., two lines in pig industry) with limited generations from the same historical population. Future research should include multiple populations, such as different selection lines or breeds. In addition, we have investigated the relationship between genetic connectedness criteria (*i*.*e*., PEVD, CD and *r*) and accuracy of prediction. However, the optimum statistical method (*i*.*e*., PEVD, CD and *r*) to measure genetic connectedness and enhance the predictive ability still remained poorly understood. Also, the level of genetic connectedness should be brought to a minimum level to ensure accurately across-herd genomic evaluation. Finally, the true genetic connectedness between populations is still unclear, which may preclude us from identifying which connectedness is the best.

## Conclusions

This study confirmed that genomic relatedness could improve the estimates of genetic connectedness across herds compared with the use of pedigree relationships. We contend that our work contributes to better understand genetic connectedness that may have a positive impact on the genomic evaluation of pig in China. Moreover, the results demonstrated the importance of the size of reference populations for genomic prediction. However, care should be taken in the design of the reference population as combined closed related populations may give less reliable result of accuracy.

## Supporting information

S1 Table Average genetic connectedness statistics between Herd1 and Herd3 in the simulation data. (DOCX)

S2 Table Accuracies of (G)EBV in the validation population when using the Herd1 or the joint reference population. (DOCX)

S3 Table Average genetic connectedness statistics between Herd1 and Herd3 in the simulation data using H matrix. (DOCX)

S4 Table Accuracies of (G)EBV in the validation population based on H matrix when using the Herd1 or the joint reference population. (DOCX)
