# Supplementary material for: The genetic connectedness calculated from genomic information and its effect on the accuracy of genomic prediction
Source: PLoS One. 2018 Jul 31;13(7):e0201400. doi: 10.1371/journal.pone.0201400 (PMC6067733; doi:10.1371/journal.pone.0201400)
Supplement: S1 Table — (DOCX) [file pone.0201400.s001.docx]

S1 Table. Average genetic connectedness statistics^1^ between Herd1 and Herd3 in the simulation data

|  |  |  | | Heritability(h^2^) | | |
| --- | --- | --- | --- | --- | --- | --- |
| ^2^No. of common sires | ^3^Methods | | ^4^Relationship matrices | 0.08 | 0.28 | 0.63 |
| 0 | PEVD | | **A^PED^** | 1.576 | 1.110 | 0.582 |
|  |  | | **G^BASE^** | 1.361 | 0.841 | 0.412 |
|  |  | | **G^0.5^(G^S^)** | 1.196(1.664) | 0.762(0.984) | 0.385(0.456) |
|  | CD | | **A^PED^** | 0.214 | 0.446 | 0.710 |
|  |  | | **G^BASE^** | 0.323 | 0.583 | 0.796 |
|  |  | | **G^0.5^(G^S^)** | 0.294(0.372) | 0.551(0.633) | 0.774(0.826) |
|  | *r_ij_* | | **A^PED^** | 0 | 0 | 0 |
|  |  | | **G^BASE^** | -0.005 | -0.006 | -0.008 |
|  |  | | **G^0.5^(G^S^)** | 0.337(0.156) | 0.435(0.238) | 0.592(0.397) |
| 1 | PEVD | | **A^PED^** | 1.573 | 1.109 | 0.581 |
|  |  | | **G^BASE^** | 1.359 | 0.839 | 0.412 |
|  |  | | **G^0.5^(G^S^)** | 1.193(1.644) | 0.759(0.974) | 0.383(0.456) |
|  | CD | | **A^PED^** | 0.215 | 0.446 | 0.709 |
|  |  | | **G^BASE^** | 0.327 | 0.584 | 0.797 |
|  |  | | **G^0.5^(G^S^)** | 0.297(0.373) | 0.552(0.632) | 0.774(0.827) |
|  | *r_ij_* | | **A^PED^** | 0 | 0.001 | 0 |
|  |  | | **G^BASE^** | -0.008 | -0.009 | -0.013 |
|  |  | | **G^0.5^(G^S^)** | 0.338(0.161) | 0.436(0.257) | 0.593(0.430) |
| 2 | PEVD | | **A^PED^** | 1.570 | 1.108 | 0.581 |
|  |  | | **G^BASE^** | 1.355 | 0.837 | 0.410 |
|  |  | | **G^0.5^(G^S^)** | 1.189(1.654) | 0.757(0.978) | 0.382(0.454) |
|  | CD | | **A^PED^** | 0.215 | 0.446 | 0.709 |
|  |  | | **G^BASE^** | 0.327 | 0.585 | 0.797 |
|  |  | | **G^0.5^(G^S^)** | 0.298(0.376) | 0.553(0.634) | 0.774(0.827) |
|  | *r_ij_* | | **A^PED^** | 0.001 | 0.001 | 0.001 |
|  |  | | **G^BASE^** | -0.007 | -0.008 | -0.012 |
|  |  | | **G^0.5^(G^S^)** | 0.339(0.160) | 0.438(0.249) | 0.595(0.429) |
| 3 | PEVD | | **A^PED^** | 1.568 | 1.107 | 0.581 |
|  |  | | **G^BASE^** | 1.353 | 0.835 | 0.410 |
|  |  | | **G^0.5^(G^S^)** | 1.188(1.664) | 0.756(0.972) | 0.382(0.453) |
|  | CD | | **A^PED^** | 0.216 | 0.446 | 0.709 |
|  |  | | **G^BASE^** | 0.328 | 0.585 | 0.797 |
|  |  | | **G^0.5^(G^S^)** | 0.298(0.378) | 0.554(0.643) | 0.775(0.827) |
|  | *r_ij_* | | **A^PED^** | 0.001 | 0.001 | 0.001 |
|  |  | | **G^BASE^** | -0.007 | -0.008 | -0.011 |
|  |  | | **G^0.5^(G^S^)** | 0.339(0.164) | 0.438(0.254) | 0.594(0.432) |
| 4 | PEVD | | **A^PED^** | 1.566 | 1.106 | 0.580 |
|  |  | | **G^BASE^** | 1.351 | 0.834 | 0.408 |
|  |  | | **G^0.5^(G^S^)** | 1.184(1.665) | 0.754(0.969) | 0.380(0.453) |
|  | CD | | **A^PED^** | 0.217 | 0.446 | 0.709 |
|  |  | | **G^BASE^** | 0.329 | 0.586 | 0.798 |
|  |  | | **G^0.5^(G^S^)** | 0.299(0.380) | 0.554(0.633) | 0.775(0.828) |
|  | *r_ij_* | | **A^PED^** | 0.002 | 0.001 | 0.001 |
|  |  | | **G^BASE^** | -0.006 | -0.007 | -0.009 |
|  |  | | **G^0.5^(G^S^)** | 0.341(0.166) | 0.440(0.251) | 0.596(0.422) |
| 5 | PEVD | | **A^PED^** | 1.564 | 1.105 | 0.580 |
|  |  | | **G^BASE^** | 1.348 | 0.833 | 0.407 |
|  |  | | **G^0.5^(G^S^)** | 1.182(1.664) | 0.753(0.965) | 0.380(0.453) |
|  | CD | | **A^PED^** | 0.217 | 0.446 | 0.709 |
|  |  | | **G^BASE^** | 0.330 | 0.586 | 0.798 |
|  |  | | **G^0.5^(G^S^)** | 0.300(0.381) | 0.554(0.633) | 0.776(0.830) |
|  | *r_ij_* | | **A^PED^** | 0.002 | 0.001 | 0.001 |
|  |  | | **G^BASE^** | -0.006 | -0.006 | -0.009 |
|  |  | | **G^0.5^(G^S^)** | 0.342(0.169) | 0.440(0.257) | 0.597(0.429) |
| 6 | PEVD | | **A^PED^** | 1.562 | 1.105 | 0.580 |
|  |  | | **G^BASE^** | 1.346 | 0.831 | 0.407 |
|  |  | | **G^0.5^(G^S^)** | 1.180(1.656) | 0.752(0.964) | 0.379(0.452) |
|  | CD | | **A^PED^** | 0.218 | 0.446 | 0.709 |
|  |  | | **G^BASE^** | 0.330 | 0.587 | 0.798 |
|  |  | | **G^0.5^(G^S^)** | 0.300(0.380) | 0.555(0.634) | 0.776(0.829) |
|  | *r_ij_* | | **A^PED^** | 0.002 | 0.002 | 0.002 |
|  |  | | **G^BASE^** | -0.006 | -0.006 | -0.008 |
|  |  | | **G^0.5^(G^S^)** | 0.343(0.169) | 0.441(0.258) | 0.597(0.429) |
| 7 | PEVD | | **A^PED^** | 1.561 | 1.104 | 0.580 |
|  |  | | **G^BASE^** | 1.345 | 0.830 | 0.406 |
|  |  | | **G^0.5^(G^S^)** | 1.179(1.642) | 0.751(0.960) | 0.379(0.450) |
|  | CD | | **A^PED^** | 0.219 | 0.447 | 0.709 |
|  |  | | **G^BASE^** | 0.331 | 0.587 | 0.798 |
|  |  | | **G^0.5^(G^S^)** | 0.301(0.379) | 0.555(0.633) | 0.776(0.829) |
|  | *r_ij_* | | **A^PED^** | 0.002 | 0.002 | 0.002 |
|  |  | | **G^BASE^** | -0.005 | -0.006 | -0.007 |
|  |  | | **G^0.5^(G^S^)** | 0.343(0.171) | 0.441(0.255) | 0.596(0.424) |
| 8 | PEVD | | **A^PED^** | 1.559 | 1.103 | 0.580 |
|  |  | | **G^BASE^** | 1.343 | 0.829 | 0.405 |
|  |  | | **G^0.5^(G^S^)** | 1.178(1.654) | 0.750(0.963) | 0.377(0.448) |
|  | CD | | **A^PED^** | 0.219 | 0.447 | 0.709 |
|  |  | | **G^BASE^** | 0.331 | 0.587 | 0.799 |
|  |  | | **G^0.5^(G^S^)** | 0.301(0.382) | 0.555(0.635) | 0.776(0.829) |
|  | *r_ij_* | | **A^PED^** | 0.003 | 0.002 | 0.002 |
|  |  | | **G^BASE^** | -0.005 | -0.005 | -0.007 |
|  |  | | **G^0.5^(G^S^)** | 0.343(0.171) | 0.441(0.255) | 0.598(0.428) |
|  |  | |  |  |  |  |
| 9 | PEVD | | **A^PED^** | 1.557 | 1.103 | 0.579 |
|  |  | | **G^BASE^** | 1.342 | 0.829 | 0.405 |
|  |  | | **G^0.5^(G^S^)** | 1.176(1.638) | 0.749(0.960) | 0.378(0.450) |
|  | CD | | **A^PED^** | 0.220 | 0.447 | 0.709 |
|  |  | | **G^BASE^** | 0.332 | 0.587 | 0.799 |
|  |  | | **G^0.5^(G^S^)** | 0.302(0.380) | 0.555(0.634) | 0.776(0.830) |
|  | *r_ij_* | | **A^PED^** | 0.003 | 0.002 | 0.002 |
|  |  | | **G^BASE^** | -0.005 | -0.005 | -0.006 |
|  |  | | **G^0.5^(G^S^)** | 0.344(0.170) | 0.442(0.258) | 0.597(0.425) |
| 10 | PEVD | | **A^PED^** | 1.555 | 1.102 | 0.579 |
|  |  | | **G^BASE^** | 1.340 | 0.828 | 0.405 |
|  |  | | **G^0.5^(G^S^)** | 1.175(1.641) | 0.748(0.964) | 0.377(0.450) |
|  | CD | | **A^PED^** | 0.221 | 0.447 | 0.709 |
|  |  | | **G^BASE^** | 0.333 | 0.588 | 0.799 |
|  |  | | **G^0.5^(G^S^)** | 0.303(0.382) | 0.556(0.636) | 0.777(0.830) |
|  | *r_ij_* | | **A^PED^** | 0.003 | 0.002 | 0.002 |
|  |  | | **G^BASE^** | -0.005 | -0.005 | -0.006 |
|  |  | | **G^0.5^(G^S^)** | 0.344(0.169) | 0.442(0.255) | 0.598(0.426) |
| 11 | PEVD | | **A^PED^** | 1.553 | 1.101 | 0.579 |
|  |  | | **G^BASE^** | 1.339 | 0.827 | 0.404 |
|  |  | | **G^0.5^(G^S^)** | 1.175(1.631) | 0.748(0.964) | 0.377(0.448) |
|  | CD | | **A^PED^** | 0.221 | 0.447 | 0.709 |
|  |  | | **G^BASE^** | 0.333 | 0.588 | 0.799 |
|  |  | | **G^0.5^(G^S^)** | 0.303(0.380) | 0.554(0.636) | 0.777(0.829) |
|  | *r_ij_* | | **A^PED^** | 0.003 | 0.002 | 0.002 |
|  |  | | **G^BASE^** | -0.004 | -0.005 | -0.006 |
|  |  | | **G^0.5^(G^S^)** | 0.343(0.167) | 0.443(0.253) | 0.598(0.424) |
| 12 | PEVD | | **A^PED^** | 1.552 | 1.101 | 0.579 |
|  |  | | **G^BASE^** | 1.337 | 0.825 | 0.403 |
|  |  | | **G^0.5^(G^S^)** | 1.173(1.634) | 0.746(0.957) | 0.376(0.445) |
|  | CD | | **A^PED^** | 0.222 | 0.448 | 0.709 |
|  |  | | **G^BASE^** | 0.333 | 0.589 | 0.799 |
|  |  | | **G^0.5^(G^S^)** | 0.304(0.382) | 0.557(0.636) | 0.777(0.829) |
|  | *r_ij_* | | **A^PED^** | 0.004 | 0.003 | 0.003 |
|  |  | | **G^BASE^** | -0.004 | -0.004 | -0.005 |
|  |  | | **G^0.5^(G^S^)** | 0.343(0.167) | 0.442(0.260) | 0.600(0.424) |
| 13 | PEVD | | **A^PED^** | 1.550 | 1.100 | 0.579 |
|  |  | | **G^BASE^** | 1.336 | 0.824 | 0.402 |
|  |  | | **G^0.5^(G^S^)** | 1.171(1.635) | 0.746(0.957) | 0.375(0.448) |
|  | CD | | **A^PED^** | 0.223 | 0.448 | 0.709 |
|  |  | | **G^BASE^** | 0.334 | 0.589 | 0.800 |
|  |  | | **G^0.5^(G^S^)** | 0.304(0.383) | 0.557(0.636) | 0.777(0.832) |
|  | *r_ij_* | | **A^PED^** | 0.004 | 0.003 | 0.003 |
|  |  | | **G^BASE^** | -0.004 | -0.004 | -0.005 |
|  |  | | **G^0.5^(G^S^)** | 0.345(0.168) | 0.443(0.260) | 0.600(0.436) |
| 14 | PEVD | | **A^PED^** | 1.549 | 1.099 | 0.579 |
|  |  | | **G^BASE^** | 1.334 | 0.824 | 0.403 |
|  |  | | **G^0.5^(G^S^)** | 1.170(1.647) | 0.745(0.952) | 0.376(0.448) |
|  | CD | | **A^PED^** | 0.223 | 0.448 | 0.709 |
|  |  | | **G^BASE^** | 0.334 | 0.589 | 0.799 |
|  |  | | **G^0.5^(G^S^)** | 0.305(0.385) | 0.557(0.634) | 0.777(0.831) |
|  | *r_ij_* | | **A^PED^** | 0.004 | 0.003 | 0.003 |
|  |  | | **G^BASE^** | -0.004 | -0.004 | -0.005 |
|  |  | | **G^0.5^(G^S^)** | 0.346(0.170) | 0.444(0.255) | 0.599(0.427) |
| 15 | PEVD | | **A^PED^** | 1.547 | 1.099 | 0.579 |
|  |  | | **G^BASE^** | 1.333 | 0.823 | 0.402 |
|  |  | | **G^0.5^(G^S^)** | 1.168(1.644) | 0.745(0.959) | 0.375(0.446) |
|  | CD | | **A^PED^** | 0.224 | 0.448 | 0.709 |
|  |  | | **G^BASE^** | 0.335 | 0.589 | 0.800 |
|  |  | | **G^0.5^(G^S^)** | 0.305(0.386) | 0.557(0.637) | 0.778(0.831) |
|  | *r_ij_* | | **A^PED^** | 0.004 | 0.003 | 0.003 |
|  |  | | **G^BASE^** | -0.004 | -0.004 | -0.004 |
|  |  | | **G^0.5^(G^S^)** | 0.346(0.170) | 0.444(0.261) | 0.600(0.430) |
| 16 | PEVD | | **A^PED^** | 1.545 | 1.098 | 0.578 |
|  |  | | **G^BASE^** | 1.331 | 0.823 | 0.402 |
|  |  | | **G^0.5^(G^S^)** | 1.166(1.639) | 0.744(0.960) | 0.374(0.445) |
|  | CD | | **A^PED^** | 0.225 | 0.448 | 0.709 |
|  |  | | **G^BASE^** | 0.336 | 0.589 | 0.800 |
|  |  | | **G^0.5^(G^S^)** | 0.306(0.386) | 0.557(0.638) | 0.777(0.830) |
|  | *r_ij_* | | **A^PED^** | 0.004 | 0.003 | 0.003 |
|  |  | | **G^BASE^** | -0.003 | -0.004 | -0.004 |
|  |  | | **G^0.5^(G^S^)** | 0.346(0.172) | 0.444(0.261) | 0.601(0.430) |
| 17 | PEVD | | **A^PED^** | 1.544 | 1.098 | 0.578 |
|  |  | | **G^BASE^** | 1.330 | 0.822 | 0.401 |
|  |  | | **G^0.5^(G^S^)** | 1.166(1.632) | 0.743(0.960) | 0.374(0.448) |
|  | CD | | **A^PED^** | 0.225 | 0.448 | 0.709 |
|  |  | | **G^BASE^** | 0.336 | 0.589 | 0.800 |
|  |  | | **G^0.5^(G^S^)** | 0.306(0.385) | 0.557(0.638) | 0.778(0.833) |
|  | *r_ij_* | | **A^PED^** | 0.004 | 0.003 | 0.003 |
|  |  | | **G^BASE^** | -0.003 | -0.003(0.261) | -0.004 |
|  |  | | **G^0.5^(G^S^)** | 0.347(0.172) | 0.445 | 0.601(0.433) |
| 18 | PEVD | | **A^PED^** | 1.542 | 1.097 | 0.578 |
|  |  | | **G^BASE^** | 1.329 | 0.821 | 0.401 |
|  |  | | **G^0.5^(G^S^)** | 1.166(1.625) | 0.743(0.957) | 0.374(0.443) |
|  | CD | | **A^PED^** | 0.226 | 0.449 | 0.709 |
|  |  | | **G^BASE^** | 0.337 | 0.590 | 0.800 |
|  |  | | **G^0.5^(G^S^)** | 0.307(0.385) | 0.558(0.638) | 0.777(0.829) |
|  | *r_ij_* | | **A^PED^** | 0.005 | 0.003 | 0.003 |
|  |  | | **G^BASE^** | -0.003 | -0.003 | -0.003 |
|  |  | | **G^0.5^(G^S^)** | 0.345(0.169) | 0.445(0.258) | 0.601(0.428) |
| 19 | PEVD | | **A^PED^** | 1.540 | 1.097 | 0.578 |
|  |  | | **G^BASE^** | 1.327 | 0.820 | 0.401 |
|  |  | | **G^0.5^(G^S^)** | 1.164(1.621) | 0.742(0.953) | 0.373(0.442) |
|  | CD | | **A^PED^** | 0.226 | 0.449 | 0.709 |
|  |  | | **G^BASE^** | 0.337 | 0.590 | 0.800 |
|  |  | | **G^0.5^(G^S^)** | 0.308(0.385) | 0.558(0.637) | 0.778(0.829) |
|  | *r_ij_* | | **A^PED^** | 0.005 | 0.003 | 0.003 |
|  |  | | **G^BASE^** | -0.003 | -0.003 | -0.003 |
|  |  | | **G^0.5^(G^S^)** | 0.346(0.172) | 0.444(0.260) | 0.602(0.428) |

^1^Standard errors for the average genetic connectedness statistics ranging from approximately 0 to 0.05

^2^Common sires = 0 represent completely disconnectedness between Herd1 and Herd3, increasing common sires increases the level of connectedness between herds.

^3^PEVD = prediction error variance of difference; *r_ij_* = prediction error correlation; CD = coefficient of determination.

^4^**A^PED^** = the usual numerator relationship matrix; **G^BASE^** = standard genomic relationship matrix; **G^0.5^** = genomic relationship matrix assuming 0.5 minor allele frequency; **G^S^** = a scaled genomic relationship matrix.
