# Supplementary material for: The genetic connectedness calculated from genomic information and its effect on the accuracy of genomic prediction
Source: PLoS One. 2018 Jul 31;13(7):e0201400. doi: 10.1371/journal.pone.0201400 (PMC6067733; doi:10.1371/journal.pone.0201400)
Supplement: S3 Table — (DOCX) [file pone.0201400.s003.docx]

S3 Table. Average genetic connectedness statistics^1^ between Herd1 and Herd3 in the simulation data using **H** matrix

|  |  |  | | Heritability(h^2^) | | |
| --- | --- | --- | --- | --- | --- | --- |
| ^2^No. of common sires | ^3^Methods | | ^4^Relationship matrices | 0.08 | 0.28 | 0.63 |
| 0 | PEVD | | **^5^H(G^BASE^)** | 1.556 | 1.085 | 0.568 |
|  | CD | | **H(G^BASE^)** | 0.224 | 0.459 | 0.716 |
|  | *r_ij_* | | **H(G^BASE^)** | 0 | 0 | 0 |
| 1 | PEVD | | **H(G^BASE^)** | 1.553 | 1.082 | 0.567 |
|  | CD | | **H(G^BASE^)** | 0.225 | 0.459 | 0.717 |
|  | *r_ij_* | | **H(G^BASE^)** | 0 | 0.001 | 0.001 |
| 2 | PEVD | | **H(G^BASE^)** | 1.549 | 1.081 | 0.566 |
|  | CD | | **H(G^BASE^)** | 0.225 | 0.459 | 0.717 |
|  | *r_ij_* | | **H(G^BASE^)** | 0.001 | 0.001 | 0.001 |
| 3 | PEVD | | **H(G^BASE^)** | 1.548 | 1.080 | 0.566 |
|  | CD | | **H(G^BASE^)** | 0.226 | 0.459 | 0.716 |
|  | *r_ij_* | | **H(G^BASE^)** | 0.002 | 0.001 | 0.001 |
| 4 | PEVD | | **H(G^BASE^)** | 1.546 | 1.080 | 0.566 |
|  | CD | | **H(G^BASE^)** | 0.227 | 0.459 | 0.716 |
|  | *r_ij_* | | **H(G^BASE^)** | 0.002 | 0.002 | 0.001 |
| 5 | PEVD | | **H(G^BASE^)** | 1.544 | 1.079 | 0.566 |
|  | CD | | **H(G^BASE^)** | 0.227 | 0.459 | 0.716 |
|  | *r_ij_* | | **H(G^BASE^)** | 0.002 | 0.002 | 0.002 |
| 6 | PEVD | | **H(G^BASE^)** | 1.542 | 1.078 | 0.566 |
|  | CD | | **H(G^BASE^)** | 0.228 | 0.459 | 0.717 |
|  | *r_ij_* | | **H(G^BASE^)** | 0.002 | 0.002 | 0.002 |
| 7 | PEVD | | **H(G^BASE^)** | 1.541 | 1.078 | 0.566 |
|  | CD | | **H(G^BASE^)** | 0.229 | 0.460 | 0.716 |
|  | *r_ij_* | | **H(G^BASE^)** | 0.003 | 0.002 | 0.002 |
| 8 | PEVD | | **H(G^BASE^)** | 1.539 | 1.077 | 0.565 |
|  | CD | | **H(G^BASE^)** | 0.229 | 0.460 | 0.717 |
|  | *r_ij_* | | **H(G^BASE^)** | 0.003 | 0.002 | 0.002 |
| 9 | PEVD | | **H(G^BASE^)** | 1.537 | 1.076 | 0.565 |
|  | CD | | **H(G^BASE^)** | 0.230 | 0.460 | 0.716 |
|  | *r_ij_* | | **H(G^BASE^)** | 0.003 | 0.002 | 0.002 |
| 10 | PEVD | | **H(G^BASE^)** | 1.535 | 1.076 | 0.565 |
|  | CD | | **H(G^BASE^)** | 0.231 | 0.460 | 0.716 |
|  | *r_ij_* | | **H(G^BASE^)** | 0.003 | 0.003 | 0.003 |
| 11 | PEVD | | **H(G^BASE^)** | 1.533 | 1.075 | 0.565 |
|  | CD | | **H(G^BASE^)** | 0.231 | 0.460 | 0.716 |
|  | *r_ij_* | | **H(G^BASE^)** | 0.004 | 0.003 | 0.003 |
| 12 | PEVD | | **H(G^BASE^)** | 1.531 | 1.074 | 0.565 |
|  | CD | | **H(G^BASE^)** | 0.232 | 0.461 | 0.716 |
|  | *r_ij_* | | **H(G^BASE^)** | 0.004 | 0.003 | 0.003 |
| 13 | PEVD | | **H(G^BASE^)** | 1.530 | 1.073 | 0.564 |
|  | CD | | **H(G^BASE^)** | 0.233 | 0.461 | 0.717 |
|  | *r_ij_* | | **H(G^BASE^)** | 0.004 | 0.003 | 0.003 |
| 14 | PEVD | | **H(G^BASE^)** | 1.529 | 1.073 | 0.564 |
|  | CD | | **H(G^BASE^)** | 0.233 | 0.461 | 0.716 |
|  | *r_ij_* | | **H(G^BASE^)** | 0.004 | 0.003 | 0.003 |
| 15 | PEVD | | **H(G^BASE^)** | 1.527 | 1.073 | 0.564 |
|  | CD | | **H(G^BASE^)** | 0.234 | 0.461 | 0.717 |
|  | *r_ij_* | | **H(G^BASE^)** | 0.004 | 0.003 | 0.003 |
| 16 | PEVD | | **H(G^BASE^)** | 1.525 | 1.072 | 0.564 |
|  | CD | | **H(G^BASE^)** | 0.234 | 0.461 | 0.716 |
|  | *r_ij_* | | **H(G^BASE^)** | 0.004 | 0.003 | 0.003 |
| 17 | PEVD | | **H(G^BASE^)** | 1.524 | 1.072 | 0.564 |
|  | CD | | **H(G^BASE^)** | 0.235 | 0.461 | 0.717 |
|  | *r_ij_* | | **H(G^BASE^)** | 0.005 | 0.004 | 0.003 |
| 18 | PEVD | | **H(G^BASE^)** | 1.521 | 1.071 | 0.564 |
|  | CD | | **H(G^BASE^)** | 0.236 | 0.462 | 0.716 |
|  | *r_ij_* | | **H(G^BASE^)** | 0.005 | 0.004 | 0.004 |
| 19 | PEVD | | **H(G^BASE^)** | 1.520 | 1.070 | 0.564 |
|  | CD | | **H(G^BASE^)** | 0.236 | 0.462 | 0.716 |
|  | *r_ij_* | | **H(G^BASE^)** | 0.005 | 0.004 | 0.004 |

^1^Standard errors for average genetic connectedness statistics ranging from approximately 0 to 0.001

^2^Common sires = 0 represent completely disconnectedness between Herd1 and Herd3, increasing common sires increases the level of connectedness between herds.

^3^PEVD = prediction error variance of difference; *r_ij_* = prediction error correlation; CD = coefficient of determination.

^4^**G^BASE^** = standard genomic relationship matrix.

^5^Very little difference in estimates occurred when **A^PED^** were combined with **G^BASE^, G^0.5^** and **G^S^**.
