# Supplementary material for: The genetic connectedness calculated from genomic information and its effect on the accuracy of genomic prediction
Source: PLoS One. 2018 Jul 31;13(7):e0201400. doi: 10.1371/journal.pone.0201400 (PMC6067733; doi:10.1371/journal.pone.0201400)
Supplement: S4 Table — (DOCX) [file pone.0201400.s004.docx]

S4 Table. Accuracies of (G)EBV in the validation population based on **H** matrix using the Herd1 or the joint reference population

| No. of common sires^1^ | Heritability | Relationship^2^ matrix | Accuracy of prediction^5^ | | |  |
| --- | --- | --- | --- | --- | --- | --- |
|  |  |  | herd1 reference^3^ | joint reference^4^ | Increase | |
| 0 |  |  |  |  |  | |
|  | 0.08 |  |  |  |  | |
|  |  | **H (G^BASE^)** | 0 | 0.26 | 0.26 | |
|  |  | **H (G^0.5^)** | 0 | 0.27 | 0.27 | |
|  |  | **H (G^S^)** | 0 | 0.26 | 0.26 | |
|  | 0.28 |  |  |  |  | |
|  |  | **H (G^BASE^)** | 0.01 | 0.42 | 0.41 | |
|  |  | **H (G^0.5^)** | 0.03 | 0.38 | 0.35 | |
|  |  | **H (G^S^)** | 0.01 | 0.42 | 0.41 | |
|  | 0.63 |  |  |  |  | |
|  |  | **H (G^BASE^)** | 0.38 | 0.51 | 0.13 | |
|  |  | **H (G^0.5^)** | 0.37 | 0.44 | 0.07 | |
|  |  | **H (G^S^)** | 0.38 | 0.51 | 0.13 | |
| 1 |  |  |  |  |  | |
|  | 0.08 |  |  |  |  | |
|  |  | **H (G^BASE^)** | 0 | 0.12 | 0.12 | |
|  |  | **H (G^0.5^)** | 0 | 0.18 | 0.18 | |
|  |  | **H (G^S^)** | 0 | 0.12 | 0.12 | |
|  | 0.28 |  |  |  |  | |
|  |  | **H (G^BASE^)** | 0 | 0.33 | 0.33 | |
|  |  | **H (G^0.5^)** | 0.03 | 0.34 | 0.31 | |
|  |  | **H (G^S^)** | 0 | 0.33 | 0.33 | |
|  | 0.63 |  |  |  |  | |
|  |  | **H (G^BASE^)** | 0.36 | 0.48 | 0.12 | |
|  |  | **H (G^0.5^)** | 0.37 | 0.41 | 0.04 | |
|  |  | **H (G^S^)** | 0.36 | 0.48 | 0.12 | |
| 2 |  |  |  |  |  | |
|  | 0.08 |  |  |  |  | |
|  |  | **H (G^BASE^)** | 0 | 0.08 | 0.08 | |
|  |  | **H (G^0.5^)** | 0 | 0.14 | 0.14 | |
|  |  | **H (G^S^)** | 0 | 0.08 | 0.08 | |
|  | 0.28 |  |  |  |  | |
|  |  | **H (G^BASE^)** | 0 | 0.28 | 0.28 | |
|  |  | **H (G^0.5^)** | 0 | 0.29 | 0.29 | |
|  |  | **H (G^S^)** | 0 | 0.28 | 0.28 | |
|  | 0.63 |  |  |  |  | |
|  |  | **H (G^BASE^)** | 0.36 | 0.48 | 0.12 | |
|  |  | **H (G^0.5^)** | 0.38 | 0.40 | 0.02 | |
|  |  | **H (G^S^)** | 0.36 | 0.48 | 0.12 | |
| 3 |  |  |  |  |  | |
|  | 0.08 |  |  |  |  | |
|  |  | **H (G^BASE^)** | 0 | 0.06 | 0.06 | |
|  |  | **H (G^0.5^)** | 0 | 0.13 | 0.13 | |
|  |  | **H (G^S^)** | 0 | 0.06 | 0.06 | |
|  | 0.28 |  |  |  |  | |
|  |  | **H (G^BASE^)** | 0 | 0.32 | 0.32 | |
|  |  | **H (G^0.5^)** | 0.03 | 0.33 | 0.30 | |
|  |  | **H (G^S^)** | 0 | 0.32 | 0.32 | |
|  | 0.63 |  |  |  |  | |
|  |  | **H (G^BASE^)** | 0.34 | 0.47 | 0.13 | |
|  |  | **H (G^0.5^)** | 0.37 | 0.43 | 0.06 | |
|  |  | **H (G^S^)** | 0.34 | 0.47 | 0.13 | |
| 4 |  |  |  |  |  | |
|  | 0.08 |  |  |  |  | |
|  |  | **H (G^BASE^)** | 0 | 0.06 | 0.06 | |
|  |  | **H (G^0.5^)** | 0 | 0.12 | 0.12 | |
|  |  | **H (G^S^)** | 0 | 0.06 | 0.06 | |
|  | 0.28 |  |  |  |  | |
|  |  | **H (G^BASE^)** | 0 | 0.26 | 0.26 | |
|  |  | **H (G^0.5^)** | 0 | 0.29 | 0.29 | |
|  |  | **H (G^S^)** | 0 | 0.26 | 0.26 | |
|  | 0.63 |  |  |  |  | |
|  |  | **H (G^BASE^)** | 0.36 | 0.48 | 0.12 | |
|  |  | **H (G^0.5^)** | 0.38 | 0.40 | 0.02 | |
|  |  | **H (G^S^)** | 0.36 | 0.48 | 0.12 | |
| 5 |  |  |  |  |  | |
|  | 0.08 |  |  |  |  | |
|  |  | **H (G^BASE^)** | 0 | 0.03 | 0.03 | |
|  |  | **H (G^0.5^)** | 0 | 0.09 | 0.09 | |
|  |  | **H (G^S^)** | 0 | 0.03 | 0.03 | |
|  | 0.28 |  |  |  |  | |
|  |  | **H (G^BASE^)** | 0 | 0.25 | 0.25 | |
|  |  | **H (G^0.5^)** | 0 | 0.28 | 0.28 | |
|  |  | **H (G^S^)** | 0 | 0.25 | 0.25 | |
|  | 0.63 |  |  |  |  | |
|  |  | **H (G^BASE^)** | 0.36 | 0.49 | 0.13 | |
|  |  | **H (G^0.5^)** | 0.37 | 0.40 | 0.03 | |
|  |  | **H (G^S^)** | 0.36 | 0.49 | 0.13 | |
| 6 |  |  |  |  |  | |
|  | 0.08 |  |  |  |  | |
|  |  | **H (G^BASE^)** | 0 | 0.01 | 0.01 | |
|  |  | **H (G^0.5^)** | 0 | 0.07 | 0.07 | |
|  |  | **H (G^S^)** | 0 | 0.01 | 0.01 | |
|  | 0.28 |  |  |  |  | |
|  |  | **H (G^BASE^)** | 0 | 0.24 | 0.24 | |
|  |  | **H (G^0.5^)** | 0 | 0.28 | 0.28 | |
|  |  | **H (G^S^)** | 0 | 0.24 | 0.24 | |
|  | 0.63 |  |  |  |  | |
|  |  | **H (G^BASE^)** | 0.36 | 0.48 | 0.12 | |
|  |  | **H (G^0.5^)** | 0.37 | 0.40 | 0.03 | |
|  |  | **H (G^S^)** | 0.36 | 0.48 | 0.12 | |
| 7 |  |  |  |  |  | |
|  | 0.08 |  |  |  |  | |
|  |  | **H (G^BASE^)** | 0 | 0.02 | 0.02 | |
|  |  | **H (G^0.5^)** | 0 | 0.08 | 0.08 | |
|  |  | **H (G^S^)** | 0 | 0.02 | 0.02 | |
|  | 0.28 |  |  |  |  | |
|  |  | **H (G^BASE^)** | 0 | 0.24 | 0.24 | |
|  |  | **H (G^0.5^)** | 0 | 0.28 | 0.28 | |
|  |  | **H (G^S^)** | 0 | 0.24 | 0.24 | |
|  | 0.63 |  |  |  |  | |
|  |  | **H (G^BASE^)** | 0.33 | 0.47 | 0.14 | |
|  |  | **H (G^0.5^)** | 0.37 | 0.42 | 0.07 | |
|  |  | **H (G^S^)** | 0.33 | 0.47 | 0.14 | |
| 8 |  |  |  |  |  | |
|  | 0.08 |  |  |  |  | |
|  |  | **H (G^BASE^)** | 0 | 0.02 | 0.02 | |
|  |  | **H (G^0.5^)** | 0 | 0.08 | 0.08 | |
|  |  | **H (G^S^)** | 0 | 0.02 | 0.02 | |
|  | 0.28 |  |  |  |  | |
|  |  | **H (G^BASE^)** | 0 | 0.30 | 0.30 | |
|  |  | **H (G^0.5^)** | 0.02 | 0.32 | 0.30 | |
|  |  | **H (G^S^)** | 0 | 0.30 | 0.30 | |
|  | 0.63 |  |  |  |  | |
|  |  | **H (G^BASE^)** | 0.36 | 0.48 | 0.12 | |
|  |  | **H (G^0.5^)** | 0.38 | 0.39 | 0.01 | |
|  |  | **H (G^S^)** | 0.36 | 0.48 | 0.12 | |
| 9 |  |  |  |  |  | |
|  | 0.08 |  |  |  |  | |
|  |  | **H (G^BASE^)** | 0 | 0.01 | 0.01 | |
|  |  | **H (G^0.5^)** | 0 | 0.07 | 0.07 | |
|  |  | **H (G^S^)** | 0 | 0.01 | 0.01 | |
|  | 0.28 |  |  |  |  | |
|  |  | **H (G^BASE^)** | 0 | 0.23 | 0.23 | |
|  |  | **H (G^0.5^)** | 0 | 0.27 | 0.27 | |
|  |  | **H (G^S^)** | 0 | 0.23 | 0.23 | |
|  | 0.63 |  |  |  |  | |
|  |  | **H (G^BASE^)** | 0.33 | 0.41 | 0.08 | |
|  |  | **H (G^0.5^)** | 0.37 | 0.46 | 0.09 | |
|  |  | **H (G^S^)** | 0.33 | 0.41 | 0.08 | |
| 10 |  |  |  |  |  | |
|  | 0.08 |  |  |  |  | |
|  |  | **H (G^BASE^)** | 0 | 0.01 | 0.01 | |
|  |  | **H (G^0.5^)** | 0 | 0.07 | 0.07 | |
|  |  | **H (G^S^)** | 0 | 0.01 | 0.01 | |
|  | 0.28 |  |  |  |  | |
|  |  | **H (G^BASE^)** | 0 | 0.23 | 0.23 | |
|  |  | **H (G^0.5^)** | 0 | 0.27 | 0.27 | |
|  |  | **H (G^S^)** | 0 | 0.23 | 0.23 | |
|  | 0.63 |  |  |  |  | |
|  |  | **H (G^BASE^)** | 0.33 | 0.46 | 0.13 | |
|  |  | **H (G^0.5^)** | 0.37 | 0.41 | 0.04 | |
|  |  | **H (G^S^)** | 0.33 | 0.46 | 0.13 | |
| 11 |  |  |  |  |  | |
|  | 0.08 |  |  |  |  | |
|  |  | **H (G^BASE^)** | 0 | 0.05 | 0.05 | |
|  |  | **H (G^0.5^)** | 0 | 0.16 | 0.16 | |
|  |  | **H (G^S^)** | 0 | 0.05 | 0.05 | |
|  | 0.28 |  |  |  |  | |
|  |  | **H (G^BASE^)** | 0 | 0.23 | 0.23 | |
|  |  | **H (G^0.5^)** | 0 | 0.27 | 0.27 | |
|  |  | **H (G^S^)** | 0 | 0.23 | 0.23 | |
|  | 0.63 |  |  |  |  | |
|  |  | **H (G^BASE^)** | 0.33 | 0.47 | 0.14 | |
|  |  | **H (G^0.5^)** | 0.37 | 0.42 | 0.07 | |
|  |  | **H (G^S^)** | 0.33 | 0.47 | 0.14 | |
| 12 |  |  |  |  |  | |
|  | 0.08 |  |  |  |  | |
|  |  | **H (G^BASE^)** | 0 | 0.04 | 0.04 | |
|  |  | **H (G^0.5^)** | 0 | 0.11 | 0.11 | |
|  |  | **H (G^S^)** | 0 | 0.04 | 0.04 | |
|  | 0.28 |  |  |  |  | |
|  |  | **H (G^BASE^)** | 0 | 0.26 | 0.26 | |
|  |  | **H (G^0.5^)** | 0.01 | 0.30 | 0.29 | |
|  |  | **H (G^S^)** | 0 | 0.26 | 0.26 | |
|  | 0.63 |  |  |  |  | |
|  |  | **H (G^BASE^)** | 0.35 | 0.48 | 0.13 | |
|  |  | **H (G^0.5^)** | 0.37 | 0.40 | 0.03 | |
|  |  | **H (G^S^)** | 0.35 | 0.48 | 0.13 | |
| 13 |  |  |  |  |  | |
|  | 0.08 |  |  |  |  | |
|  |  | **H (G^BASE^)** | 0 | 0.02 | 0.02 | |
|  |  | **H (G^0.5^)** | 0 | 0.07 | 0.07 | |
|  |  | **H (G^S^)** | 0 | 0.02 | 0.02 | |
|  | 0.28 |  |  |  |  | |
|  |  | **H (G^BASE^)** | 0 | 0.25 | 0.25 | |
|  |  | **H (G^0.5^)** | 0 | 0.30 | 0.30 | |
|  |  | **H (G^S^)** | 0 | 0.25 | 0.25 | |
|  | 0.63 |  |  |  |  | |
|  |  | **H (G^BASE^)** | 0.30 | 0.40 | 0.10 | |
|  |  | **H (G^0.5^)** | 0.33 | 0.37 | 0.04 | |
|  |  | **H (G^S^)** | 0.30 | 0.40 | 0.10 | |
| 14 |  |  |  |  |  | |
|  | 0.08 |  |  |  |  | |
|  |  | **H (G^BASE^)** | 0 | 0 | 0 | |
|  |  | **H (G^0.5^)** | 0 | 0.05 | 0.05 | |
|  |  | **H (G^S^)** | 0 | 0 | 0 | |
|  | 0.28 |  |  |  |  | |
|  |  | **H (G^BASE^)** | 0 | 0.21 | 0.21 | |
|  |  | **H (G^0.5^)** | 0 | 0.27 | 0.27 | |
|  |  | **H (G^S^)** | 0 | 0.21 | 0.21 | |
|  | 0.63 |  |  |  |  | |
|  |  | **H (G^BASE^)** | 0.33 | 0.47 | 0.14 | |
|  |  | **H (G^0.5^)** | 0.37 | 0.42 | 0.07 | |
|  |  | **H (G^S^)** | 0.33 | 0.47 | 0.14 | |
| 15 |  |  |  |  |  | |
|  | 0.08 |  |  |  |  | |
|  |  | **H (G^BASE^)** | 0 | 0 | 0 | |
|  |  | **H (G^0.5^)** | 0 | 0.05 | 0.05 | |
|  |  | **H (G^S^)** | 0 | 0 | 0 | |
|  | 0.28 |  |  |  |  | |
|  |  | **H (G^BASE^)** | 0 | 0.20 | 0.20 | |
|  |  | **H (G^0.5^)** | 0 | 0.26 | 0.26 | |
|  |  | **H (G^S^)** | 0 | 0.20 | 0.20 | |
|  | 0.63 |  |  |  |  | |
|  |  | **H (G^BASE^)** | 0.30 | 0.44 | 0.14 | |
|  |  | **H (G^0.5^)** | 0.33 | 0.37 | 0.04 | |
|  |  | **H (G^S^)** | 0.30 | 0.44 | 0.14 | |
| 16 |  |  |  |  |  | |
|  | 0.08 |  |  |  |  | |
|  |  | **H (G^BASE^)** | 0 | 0 | 0 | |
|  |  | **H (G^0.5^)** | 0 | 0.05 | 0.05 | |
|  |  | **H (G^S^)** | 0 | 0 | 0 | |
|  | 0.28 |  |  |  |  | |
|  |  | **H (G^BASE^)** | 0 | 0.20 | 0.20 | |
|  |  | **H (G^0.5^)** | 0 | 0.26 | 0.26 | |
|  |  | **H (G^S^)** | 0 | 0.20 | 0.20 | |
|  | 0.63 |  |  |  |  | |
|  |  | **H (G^BASE^)** | 0.35 | 0.48 | 0.13 | |
|  |  | **H (G^0.5^)** | 0.37 | 0.40 | 0.03 | |
|  |  | **H (G^S^)** | 0.35 | 0.48 | 0.13 | |
| 17 |  |  |  |  |  | |
|  | 0.08 |  |  |  |  | |
|  |  | **H (G^BASE^)** | 0 | 0 | 0 | |
|  |  | **H (G^0.5^)** | 0 | 0.05 | 0.05 | |
|  |  | **H (G^S^)** | 0 | 0 | 0 | |
|  | 0.28 |  |  |  |  | |
|  |  | **H (G^BASE^)** | 0 | 0.19 | 0.19 | |
|  |  | **H (G^0.5^)** | 0 | 0.26 | 0.26 | |
|  |  | **H (G^S^)** | 0 | 0.19 | 0.19 | |
|  | 0.63 |  |  |  |  | |
|  |  | **H (G^BASE^)** | 0.30 | 0.44 | 0.14 | |
|  |  | **H (G^0.5^)** | 0.30 | 0.37 | 0.07 | |
|  |  | **H (G^S^)** | 0.30 | 0.44 | 0.14 | |
| 18 |  |  |  |  |  | |
|  | 0.08 |  |  |  |  | |
|  |  | **H (G^BASE^)** | 0 | 0.03 | 0.03 | |
|  |  | **H (G^0.5^)** | 0 | 0.09 | 0.09 | |
|  |  | **H (G^S^)** | 0 | 0.03 | 0.03 | |
|  | 0.28 |  |  |  |  | |
|  |  | **H (G^BASE^)** | 0 | 0.19 | 0.19 | |
|  |  | **H (G^0.5^)** | 0 | 0.26 | 0.26 | |
|  |  | **H (G^S^)** | 0 | 0.19 | 0.19 | |
|  | 0.63 |  |  |  |  | |
|  |  | **H (G^BASE^)** | 0.35 | 0.49 | 0.14 | |
|  |  | **H (G^0.5^)** | 0.37 | 0.40 | 0.03 | |
|  |  | **H (G^S^)** | 0.35 | 0.49 | 0.14 | |
| 19 |  |  |  |  |  | |
|  | 0.08 |  |  |  |  | |
|  |  | **H (G^BASE^)** | 0 | 0.02 | 0.02 | |
|  |  | **H (G^0.5^)** | 0 | 0.07 | 0.07 | |
|  |  | **H (G^S^)** | 0 | 0.02 | 0.02 | |
|  | 0.28 |  |  |  |  | |
|  |  | **H (G^BASE^)** | 0 | 0.24 | 0.24 | |
|  |  | **H (G^0.5^)** | 0 | 0.29 | 0.29 | |
|  |  | **H (G^S^)** | 0 | 0.24 | 0.24 | |
|  | 0.63 |  |  |  |  | |
|  |  | **H (G^BASE^)** | 0.35 | 0.49 | 0.14 | |
|  |  | **H (G^0.5^)** | 0.37 | 0.40 | 0.03 | |
|  |  | **H (G^S^)** | 0.35 | 0.49 | 0.14 | |

^1^Common sires=0 represent completely disconnectedness between Herd1 and Herd3, increasing common sires increases the level of connectedness between herds.

^2^**G^BASE^** = standard genomic relationship matrix; **G^0.5^** = genomic relationship matrix assuming 0.5 minor allele frequency; **G^S^** = a scaled genomic relationship matrix.

^3^Herd1 reference: reference population consisting of only individuals from Herd1.

^4^Joint reference: reference population consisting of individuals from both Herd1 and Herd3.

^5^Standard errors for accuracy of prediction ranging from approximately 0.021 to 0.117.
